# Dopamine receptor antagonists effects on low-dimensional attractors of local field potentials in optogenetic mice

**DOI:** 10.1371/journal.pone.0223469

**Published:** 2019-10-16

**Authors:** Sorinel A. Oprisan, Xandre Clementsmith, Tamas Tompa, Antonieta Lavin

**Affiliations:** 1 Department of Physics and Astronomy, College of Charleston, Charleston, SC, United States of America; 2 Department of Computer Science, College of Charleston, Charleston, SC, United States of America; 3 Department of Neuroscience, Medical University of South Carolina, Charleston, SC, United States of America; 4 Faculty of Healthcare, Department of Preventive Medicine, University of Miskolc, Miskolc, Hungary; Georgia State University, UNITED STATES

## Abstract

The goal of this study was to investigate the effects of acute cocaine injection or dopamine (DA) receptor antagonists on the medial prefrontal cortex (mPFC) gamma oscillations and their relationship to short term neuroadaptation that may mediate addiction. For this purpose, optogenetically evoked local field potentials (LFPs) in response to a brief 10 ms laser light pulse were recorded from 17 mice. D1-like receptor antagonist SCH 23390 or D2-like receptor antagonist sulpiride, or both, were administered either before or after cocaine. A Euclidian distance-based dendrogram classifier separated the 100 trials for each animal in disjoint clusters. When baseline and DA receptor antagonists trials were combined in a single trial, a minimum of 20% overlap occurred in some dendrogram clusters, which suggests a possible common, invariant, dynamic mechanism shared by both baseline and DA receptor antagonists data. The delay-embedding method of neural activity reconstruction was performed using the correlation time and mutual information to determine the lag/correlation time of LFPs and false nearest neighbors to determine the embedding dimension. We found that DA receptor antagonists applied before cocaine cancels out the effect of cocaine and leaves the lag time distributions at baseline values. On the other hand, cocaine applied after DA receptor antagonists shifts the lag time distributions to longer durations, i.e. increase the correlation time of LFPs. Fourier analysis showed that a reasonable accurate decomposition of the LFP data can be obtained with a relatively small (less than ten) Fourier coefficients.

## Introduction

Use of psychostimulants, such as cocaine, is a serious health problem and opens the door to neurobiological changes in limbic and cortical circuits that engage cognitive and emotive processing. We have just began to understand the cellular adaptations that occur in the cortex following a single exposure to cocaine and their contribution to the continuous and further use of drugs of abuse. The behavioral consequences of first time cocaine use are varied and appear to be somewhat contradictory. First time cocaine users often report feeling a sharpening of the senses [1], and anecdotal information suggest that acute cocaine increases attention. Indeed, individuals with ADHD will sometimes self-medicate with cocaine [2]. Contrastingly, Jentsch and colleagues [3] have shown that acute cocaine administration impairs performance on a reversal learning task, and several studies have reported compromised performance during repeated acquisition tasks in monkeys [4, 5]. Additionally, imaging studies in humans have shown that acute cocaine administration induces prominent activation of the prefrontal cortex, primarily in the dorsolateral regions [6]. Furthermore, acute cocaine administration has been linked to poor impulse control [3, 7, 8]. Therefore, it seems that first time cocaine use may give users a sense of enhanced awareness, while cognitive performance is diminished. Cocaine abuse is a public health challenge for which we are still seeking more effective treatments [9, 10]. Additionally, patients relapse in greater numbers increasing the social and economic cost of this disease and the burden on medical and welfare systems. Thus, understanding the cellular and network process underlying the effects of cocaine in the prefrontal cortex is a critical necessity.

Neuronal oscillations are thought to be general and fundamental mechanisms for enabling coordinated activity during normal brain functions [11–13] and it is within this framework that the function of gamma oscillations have recently acquired importance. Gamma oscillations in the cortex involve the reciprocal interaction between interneurons, mainly PV+ fast spiking interneurons (FS PV+) and principal cells [14]. Gamma oscillations appear to be a critical mechanism underlying the cognitive and behavioral function of mPFC. It is therefore highly likely that gamma oscillations in mPFC would be altered by cocaine administration. In this study we investigated changes in gamma oscillations following an acute administration of cocaine and the effects of selective dopamine (DA) receptor antagonist on the gamma band oscillations.

### Parvalbumin (PV) interneurons and pyramidal cells in the mPFC

The vast majority (over 70% according to [15, 16]) of excitatory neural population is made of pyramidal cells. Pyramidal cells span cortical layers 2-6 [16] and are thought to be involved in both maintaining the working memory [17] and the UP states of persistent network activity [18, 19]. The main modulators of the pyramidal cells are interneurons, of which PV+ fast-spiking interneurons represent the majority [20–22]. PV+ neurons coordinate the output of the local minicolumns [23, 24], maintain and/or modulate both the 15–30 Hz beta [25–27] and the 25–40 Hz gamma [28–30] rhythms of the brain, and facilitate information processing [31–33]. PV+ interneurons are known to both modulate the firing rate of pyramidal cells through their dendritic projections [34, 35] and to block action potentials through their axonal projections [34–38]. Abnormal activity of PV+ inerneurons have been linked to autism [39–42], schizophrenia [32, 43–45], sensory hypersensitvity and neural hyper-excitability [46–49].

### Optogenetic studies of mPFC

Optogenetics refers to the use of optical stimuli to elicit neural responses from neurons whose ionic channels were genetically modified to respond to light stimuli. Optical fibers transmit the light stimulus from the external laser to the brain region of the mouse. For optogenetics, the optical fiber is integrated with electrophysiological probes and form a device called an optrode [50]. In basic science studies, optogentics has been used for investigating neural plasticity mechanisms [51–53], understanding information processing by neural networks [29, 30, 54], and testing hippocampal memory formation hypotheses [55, 56]. In behavioral studies, optogentics proved invaluable in studying feeding [57–60], fear conditioning [61, 62], and aggression [63]. The ultimate goal of these studies is to find optogenetics-based solutions to neural disorders, such as anxiety and depression [64–66], and ameliorate neurodegenerative conditions, such as Parkinson disease [67, 68], and epilepsy [69–72]. Finally, another promising development includes the use of optogentics for the restoration of visual functions in blind animals [73–75] and the design of neural interface [76].

### Dopamine (DA) receptor

The widely-accepted paradigm regarding the mechanisms of cocaine and amphetamine effect is through increasing synaptic levels of DA, mainly through DA transporter’s inhibition [77–80]. As a result, DA receptors are exposed to an elevated and sustained levels of endogenous DA, which amounts to an indirect DA agonist effect [81]. All drugs of abuse produce increased levels of DA [82–85]. Additionally, the relapse to cocaine-seeking behavior implicates the mesolimbic DA system [86–89] through DA receptor’s potentiation [86, 90].

There are several kinds of DA receptors, which are studied with the purpose of finding effective addiction treatments [91–93]. Five DA receptor subtypes, called D1-D5, have been identified [94–97], although they appear to form two classes of pharmacologically and biochemically similar receptors, i.e. D1-like receptors, which refer to D1/D5, and D2-like receptors, which refer to D2/D3/D4, respectively. Both D1- and D2-like receptors have both been implicated in the discriminative and reinforcing effects of cocaine [78, 98, 99], although they seem to be somewhat selective in their response to cocaine [100, 101].

We previously carried out a similar computational study on *a subset of the same animals* under control conditions [102] and following an acute cocaine injection [103]. The present study expands our previous investigation of cocaine effects on mPFC [102, 103] in which we systemically injected DA receptor antagonists & cocaine. As in our previous studies, we corrected for the light-induced phase resetting using both the autocorrelation [102, 104] and Hilbert’s transform [105]. Delay-embedding method has been used for estimating the delay (lag) time and embedding dimension of the LFPs [102, 106, 107]. We also used spectral analysis to model the LFPs and found that a significant fraction of the data trend can be captured with as few as ten Fourier coefficients.

## Materials and methods

A detailed description of the procedures can be found in the previous two papers of this series [102, 103]. Mice Cre-PV+ adult males were mounted in an steretoaxic apparatus, and a small hole drilled over the PFC to insert the optrodes. Temperature was maintained at 35 C via a thermal blanket and isoflourane anesthesia was maintained using an isoflourane anesthesia system. At the end of the recordings animals were deeply anesthetized and the brain extracted to confirm anatomical localization of optrodes. All procedures were done in accordance to the National Institute of Health guidelines as approved by the Medical University of South Carolina Institutional Animal Care and Use Committee.

### Experimental protocol

The response of medial prefrontal cortex (mPFC) to a brief 10 ms optical stimulation was measured from 2-s long LFP recordings that were repeated 100 times for each of the 17 animals in this study. The fast-spiking interneurons were activated by laser stimulation. This study used the same mice model together with optogenetics and in vivo electrophysiology described in detail in [108]. The viral vector (AAV2/5. EF1a. DIO. hChR2(H134R)—EYFP. WPRE. hGH, Penn Vector Core, University of Pennsylvania) has been injected to the mPFC of male mice at least 3 weeks before electrophysiological recordings (B6; 129P2 − Pval^*btm*1(*Cre*)*Arbr*/*J*^ Jackson Laboratory (Bar Harbor, ME, USA) [108]. The extracellular LFPs were amplified (Grass Technologies, West Warwick, RI, USA), digitized at 10 kHz (1401plus data acquisition system), and analyzed offline. The signal was filtered (Quest Scientific Inc., Canada) and band-pass online between 0.1 and 130 Hz to obtain the LFPs. The light stimulation was provided by a 473 nm laser (DPSS Laser System, OEM Laser Systems Inc, East Lansing, MI, USA).

The animals received acute systemic injection of the D1 receptor antagonist SCH 23390, 1.0 mg/kg, i.p. or the D2 receptor antagonist sulpiride, 15 mg/kg, i.p. and optical stimulation.

The novelty of this study compared to others involving DA receptor antagonists is the use of delay embedding for data visualization and spectral analysis for data modeling. The novelty of this study compared to the previous two [102, 103] is that we targeted DA receptors with specific receptor antagonists. When the DA receptor antagonist was injected systemically after the baseline and prior to cocaine the name of the trial is *xxxbyy* where *xxx* is either *sch* for SCH 23390 or *sulp* for sulpiride, the letter *b* stands for *before* cocaine and *yy* is the mouse number. For example, *schb*14 means LFP recordings from mouse # 14 with SCH 23390 D1 receptor antagonist applied before cocaine. Similarly, the name *xxxayy* means the receptor antagonist *xxx* was applied *after* cocaine. Finally, in other trials we applied both DA receptor antagonists together and the name of the trial is *both* followed by either *a* (after cocaine) or *b* (before cocaine) and the mouse number (see Table 1). For example, *bothb*24 means LFP recordings from mouse # 24 with SCH 23390 & sulpiride applied before cocaine.

**Table 1:** Experimental protocol for DA receptor antagonists.

Mouse #	cocaine first	SCH23390	sulpiride	cocaine last	trial name
8			x	x	sulpb8
9			x	x	sulpb9
10			x	x	sulpb10
11			x	x	sulpb11
12		x		x	schb12
13		x		x	schb13
14		x		x	schb14
15		x		x	schb15
16	x	x			scha16
17	x		x		sulpa17
18	x		x		sulpa18
19	x		x		sulpa19
20		x	x	x	bothb20
21		x	x	x	bothb21
22		x	x	x	bothb22
23	x	x	x		botha23
24	x	x			scha24

In the following, we briefly summarize the main methods used here for data analysis, visualization, and modeling.

### Cluster analysis with dendrograms

**Fig 1:**
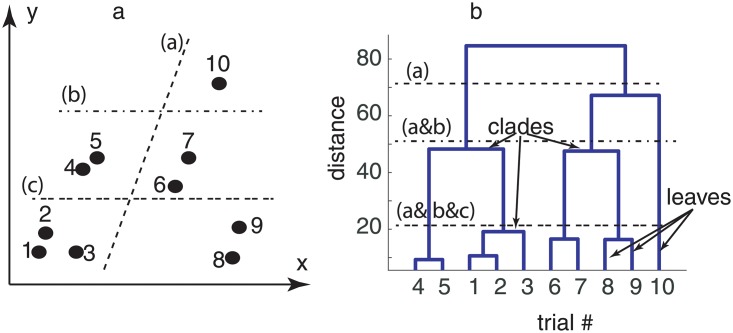
Dendrogram analysis. Euclidian distance between scattered two-dimensional data (a) was used for assigning individual points to clusters based on their similarity as measured by the Euclidian distance between data points (b). The vertical axis of the dendrogram (b) gives the distance between different data points (leaves). The closest points #4 and #5 (see panel a) are clustered together in the dendrogram (panel b) with the lowest horizontal linkage (distance). A horizontal dashed line marked with (a) through the dendrogram (panel b) shows two clusters, which are identified in the actual data (panel a) by the corresponding dashed line. Lower maximum distances among data points split the data in three (dashed-dotted line marked with (b)) and five (double dashed line marked with (c)) clusters, respectively.

A dendrogram is a diagram that shows the hierarchical relationship between objects. The dendrograms are used for allocating objects to clusters. For example, a scattered plot in a two-dimensional Euclidian space may look like Fig 1a. The (*x*, *y*) pair could be single measurements data or, as in our study, they could represent an entire trial. In our example, the dendrogram computed the “distance” between the scattered points shown in Fig 1a and then ordered them according to the distance, which is shown along the vertical axis of the dendrogram in Fig 1b. In the example above, we can immediately notice from Fig 1a that data points #4 and #5 are the closest across all ten, and therefore they will be shown in the dendrogram of Fig 1b as the most similar, i.e. the height of the link that joins them together is the smallest. A cursory inspection of Fig 1a suggests that the next closest data points are #1 and #2, which translates into the next most similar objects in the dendrogram shown in Fig 1b. In a dendrogram, the height of the dendrogram indicates the order in which the clusters were joined. Each joining (fusion) of two clusters is represented on the dendrogram by the splitting of a vertical line into two vertical lines. The vertical position of the split, shown by the short horizontal bar called clade, gives the distance (dissimilarity) between the two clusters. The terminal end of each clade is called a leaf.

Observations are allocated to clusters by drawing a horizontal line through the dendrogram. In our example, below the dashed line marked (a) in Fig 1b there are two clusters, which are separated by the corresponding dashed line in Fig 1a. If the required maximum distance is lowered (see dashed-dotted line marked (b) in Fig 1b), then we identify three clusters, which correspond to separated data clusters in Fig 1a. By further decreasing the required maximum distance within a cluster (see the double dashed line marked (c) in Fig 1b), then five clusters emerge.

Although in our example, we simply used the Euclidian distance between the points shown in Fig 1a, other distance measures could be used for hierarchical organization of data. Among many possible distance measures, the correlation coefficient [109] and the Euclidian distance [110] are most common. For example, a correlation-based distance is *distance* = 1 − *c*, where *c* is the correlation coefficient (*c*).

### Delay-embedding method

In this paper series (see also [102, 103]), we used the delay-embedding method of nonlinear dynamics to estimate the number of degrees of freedom of the steady activity of mPFC neural network. The challenge is to extract, or reconstruct, from the one-dimensional data (time series) the actual multidimensional attractors of neural activity.

Theoretically, the first derivative of continuous functions, or equivalently the first order difference of discrete data sets, is orthogonal to the original time series. Therefore, one could use the original time series and its first order difference as the two “natural” dimensions (axes) to unfold the dynamics of the system. If the phase space trajectories cross each other, then we could use the second derivative as the third orthogonal direction in the phase space and try to unfold the attractor again. The procedure could be repeated until all self-crossings of the phases space trajectory are eliminated, which is the final unfolded attractor of neural dynamics [111]. Takens’ theorem proved that for regularly sampled and noise free time series, the attractor reconstructed using the above-described differential embedding is diffeomorphic to the original (unknown) attractor [112, 113].

A more robust to noise approach is to use the delay-embedding method [112, 114], which takes a time series *x*_*i*_ = *x*(*i*Δ*t*) with *i* = 1, 2, …,*N* where *N* is the number of data points and Δ*t* is the uniform sampling time, and expands it into a *d*−dimensional vector by temporally shifting the data [112, 114–116]:

$$\begin{array}{c} x_{i}=\left(x_{i},\;x_{i+n},\ldots ,x_{i+(d-1)n}\right), \end{array}$$

where *τ* = *n*Δ*t* is the delay, or lag, time. The challenge of the delay-embedding method is to correctly select both the number of independent variables, i.e. the embedding dimension *d*, and the lag time *τ*.

#### The lag time

The challenge of selecting the “right” delay time is to avoid both the *redundancy*, i.e. high correlation among the data points because of a too small delay time, and completely de-correlating the data points by a too large delay time [117]. As in the previous studies [102, 103], we used both the autocorrelation [117–122] and the average mutual information (AMI) [123–125] for estimating the lag time *τ*. The first zero crossing of the autocorrelation ensures that the lag time completely de-correlated the original and the time-shifted time series [126]. While convenient, the autocorrelation method only eliminates the linear correlation. As a result, we also estimating the lag time using the first minimum of the nonlinear autocorrelation function called *Average Mutual Information* (AMI) [123, 125].

In order to allow a direct comparison of our current results on the effect of DA receptor antagonist combined with cocaine on LFP recordings against similar results on the same mice for baseline recordings (see [102]) and cocaine (see [103]), we only analyzed the lag times obtained from the autocorrelation function method. However, for completeness, we also briefly summarize the statistics of the lag times obtained from AMI.

#### The embedding dimension

As before [102, 103], we used the false nearest neighbors (FNN) algorithm [124, 127, 128] to estimate the embedding dimension. Intuitively, a high-dimensional phase space trajectories projected onto a too low dimensional embedding space has self-crossing points, or false nearest neighbors (FNN). Such FNN could be eliminated by progressively increasing the dimension of the embedding space until reaching a reasonably low percentage of FNN [127].

Without repeating all the details in [102, 103], we used the lag times computed in the previous section for each condition to estimate the embedding dimension with variable distance ratios, *f*, between 2 and 20. The distance ratio *f* estimates the factor by which the distance between two points increased when increasing the embedding dimension by one unit [126, 127, 129]. Usually, small *f* overestimates the percentage of FNN, whereas a too large *f* values give a large number of false positives. In our study, for *f* > 10, the percentage of FNN drops below 1% for an embedding dimension *d*_*E*_ = 3 (not shown), whereas for *f* > 12 the percentage of FNN dropped below 10^−6^% at *d*_*E*_ = 3.

As in the previous studies [102, 103], we further removed spurious temporal correlations [130, 131] with Theiler windows from 100 to 8000 sampling times [131, 132]. For all tested Theiler windows, the percentage of FNN dropped below 0.1% for an embedding dimension of *d*_*E*_ = 3 and *f* > 10.

### Fréchet distance method

Fréchet distance [133] relates to the “man walking a dog on a leash” paradigm, i.e. find the shortest leash needed such that the man walks along one curve and the dog walks along the other [134]. Both the man and the dog can adjust their speeds but are not allowed to move backwards [135]. Fréchet distance is a topological measure of the similarity between two curves [134], i.e. it does not consider any temporal constrains regarding the location of figurative points on the two curves. We computed Fréchet distance using the algorithm in [136].

### Fourier method

We used Fourier analysis to determine the spectral composition of each trial. Any periodic signal *x*(*t*) of period *T* could be represented as an infinite sum of harmonics [137]:

$$\begin{array}{c} x\left(t\right)=\sum_{k=-\infty }^{k=+\infty }a_{k}e^{j\omega_{k}t}=a_{0}+\sum_{k=1}^{k=\infty }2Re(a_{k}e^{j\omega_{k}t}), \end{array}$$ (1)

where $j=\sqrt{-1}$ is the imaginary unit, *a*_*k*_ are called Fourier coefficients, and *ω*_*k*_ = *kω*_0_ are k^*th*^ order harmonics of the fundamental frequency *ω*_0_ = 1/*T*. For real periodic signals in continuous time, the above Fourier representation becomes [138]:

$$\begin{array}{c} x\left(t\right)=a_{0}+2\sum_{k=1}^{k=\infty }A_{k}\cos\left(\omega_{0}kt+\theta_{k}\right), \end{array}$$ (2)

where the complex Fourier coefficients were represented in polar coordinates as $a_{k}=A_{k}e^{j\theta_{k}}$ with *A*_*k*_ the amplitude and *θ*_*k*_ the corresponding phase of *k*^*th*^ harmonic. Eq 2 is sometimes called the synthesis equation because it can be used for synthesizing a signal that resembles the original time series based on its Fourier frequency spectrum.

## Results

As in the previous papers of this series [102, 103], the response of the local mPFC neural network to a brief 10 ms light pulse was recorded for 2 s and the procedure repeated 100 times for each animal. We discarded the transient response of the neural network by removing the first 0.5 s of each trial. The transient response contains information regarding the phase resetting of the entire local neural network, which has been successfully applied to epilepsy [139, 140] or Parkinson’s [141, 142] studies. However, here we focused on the last 1.5 s of steady activity of each trial with the purpose of identifying stable and repeatable patterns of neural activity that may connect the control [102], the pure cocaine [103], and this DA receptor antagonists study.

### Phase resetting of LFPs

**Fig 2:**
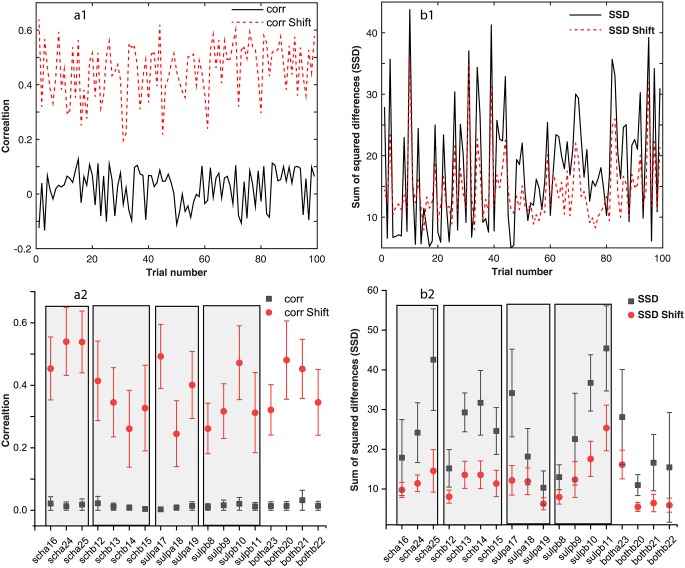
The correlation among trials. The correlation coefficients of a “reference” trial with each of the other 99 trials for a particular animal and condition (black continuous line in panel a1) are relatively low due to the random light stimulus timing relative to the network’s activity. After circularly shifting the trials, we noticed an order of magnitude increase of the correlation coefficient (see a1 dashed line). The vertical error bar corresponds to one standard deviation. Similar analysis was carried out for the sum of the squared differences (SSD) between the arbitrary “reference” trial and each of the other 99 trials for each animal and condition (see panels b1 and b2). The increase in the correlation coefficient due to phase correction also decreases the SSD values. Rectangular shaded areas mark similar conditions, such as *scha*, *schb*, etc.

A brief 10 ms light pulse alters both the phase and the amplitude of the ongoing oscillatory activity of the local mPFC [104, 106, 143, 144]. Such transient resettings dissipate after a few cycles and eventually the neural network returns to its unperturbed steady activity [105]. We corrected the trials for the transient phase resetting induced by light stimuli by circularly shifting each LFP trace with respect to an arbitrary “reference” trial until maximizing the correlation coefficient [105, 144]. Phase resetting correction led to a significant increase in the coefficient of correlation among trials as seen in Fig 2a1 for the case of *scha*16 and in Fig 2a2 for all mice and conditions. The correlation coefficients before circularly shifting the data to account for the transient LFP phase resetting has very low values (see the solid black trace in Fig 2a1 and solid black squares in Fig 2a2). For each condition, e.g. *scha*, *schb*, *supla*, *sulpb*, *botha*, *bothb*, and each animal #, the averages of all correlation coefficients (such as the one shown in panel a1) indicate a very low initial correlation (solid black squares in Fig 2a2) due to the fact that the incoming light stimulus finds the neural network at random phases. By circularly shifting the trials to maximize the correlation coefficient we corrected for the random phase of light stimulus relative to the ongoing rhythmic activity of the network (solid red circles in Fig 2a2). Although for the same animal and the same condition the correlation coefficient differs from one pair of trials to the other as shown in Fig 2a1, we averaged the correlation coefficients over all 100 trials and only showed in Fig 2a2 the average and standard deviation bar. We further averaged all data for the same condition to gain a better understanding of the average increase of correlation due to phase shifting.

For example, the three *scha*16, *scha*24, and *scha*25 data points in Fig 2a2 (already averaged over all trials) were considered as a single *scha* condition in Table 2. While the improvement in the correlation coefficient is qualitatively apparent from Fig 2, the compact numerical summary in Table 2 shows that in most cases the circular shifting improvement exceeded one order of magnitude.

**Table 2:** Averages ± standard deviations of the correlation coefficient and the sum of squared differences (SSD) before and after shifting the trials to correct for the phase resetting.

Condition	Corr.	Corr. Shift	SSD	SSD Shift
scha	0.018 ± 0.004	0.51 ± 0.05	28 ± 13	12 ± 2
schb	0.011 ± 0.008	0.34 ± 0.06	25 ± 7	12 ± 3
sulpa	0.008 ± 0.006	0.4 ± 0.1	21 ± 12	10 ± 3
sulpb	0.015 ± 0.004	0.34 ± 0.09	29 ± 14	16 ± 7
botha	0.13 ± 0.01	0.32 ± 0.08	28 ± 12	16 ± 4
bothb	0.02 ± 0.01	0.43 ± 0.07	14 ± 3	6.0 ± 0.5

We also computed the sum of squared differences (SSD) between the “reference” trial and all the other trials for each animal and each condition (see one example in Fig 2b1 for *scha*16). As expected, circularly shifting the trials to maximize the correlation coefficient also reduces the SSD among trials (Fig 2b1), overall per animal and condition (Fig 2b2), and also when aggregated in a single average per condition as in Table 2.

### Dendrograms of phase shifted LFPs

**Fig 3:**
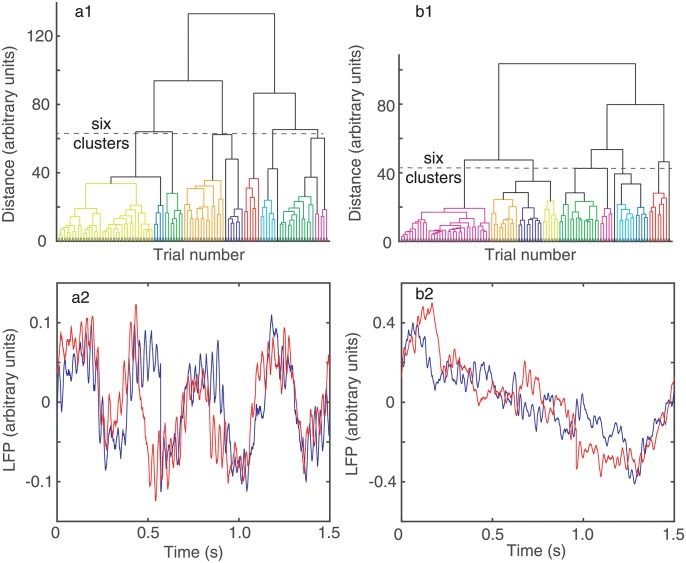
Dendrogram-based groups of similar LFPs using Euclidian distance among trials. Twelve similar clusters were formed out of the 100 trials both without (a) and with (b) circular phase shifting to account for network phase resetting due to light stimulus. The distance threshold that generates, for example, six clusters was about 62 arbitrary units without phase correction (a) and decreased to about 42 arbitrary units (b) after phase correction (see the horizontal dashed lines in a1 and b1). Panels a2 and b2 show two representative examples of LFP recordings belonging to two different clusters in the dendrogram. The recordings in panel a2 belong to the largest cluster whereas in b2 they belong to the second largest cluster in the dendrogram shown in panel b1. The LFP pattern classifier correctly separated dissimilar recordings in different clusters and also correctly placed in the same cluster similarly-looking LFPs.

A dendrogram is a visual representation of “relationships” among trials. The horizontal axis of the dendrogram in Fig 3a1–3b1 shows the trial index. However, the trials are not listed in their recording order but rather based on their membership to the respective cluster. The vertical axis of the dendrogram is the *distance* between the “leaves”, i.e. trials, of the dendrogram tree (see Fig 3a1–3b1).

We used dendrograms for two purposes: (1) to qualitatively check that the circular shifting of trials for the purpose of maximizing the correlation coefficient also reduced the distance among trials in a cluster, and (2) to quantitatively measure the amount of overlap between the baseline and the DA receptor antagonists trials.

Since we established that the correlation coefficients increase by circularly shifting the trials (see Fig 2a1 and 2a2) then it follows that the corresponding dendrogram distances would decrease (see Fig 3a1–3b1). In particular, it is expected that the distances in the circularly shifted dendrogram (Fig 3b1) should be smaller compared to the original data (Fig 3a1). The reason we used the dendrogram of the circularly shifted data is because it better separated trials in disjoint classes as we can see from two examples of LFP traces shown Fig 3a2 and 3b2. Indeed, before circularly shifting the data, even trials as dissimilar-looking as those shown in panels a2 and b2 could be classified as belonging to the same cluster in Fig 3a1. This is because a certain amount of correlation, no matter how small, would still exist between two trials recorded from the same system even if they have a random phase difference between them due to light stimulus resetting of neural activity. By circularly shifting the trials with respect to one (reference) trial to maximize the correlation coefficient, we eliminated random overlaps among trials which resulted in small correlations shown in Fig 2a1 and 2a2 and only retained for further analysis correlations that are significant, i.e. one order of magnitude larger than before circularly shifting the data.

The Euclidian distance that measures the similarities among the “leaf” nodes of the dendrogram (see Fig 3a1 and 3b1) has been significantly reduced by phase reseting correction. For example, for the *scha*16 experiment the distance threshold for breaking the 100 trials of into six clusters was reduced from 62 (arbitrary) units (see the horizontal dashed line in Fig 3a1) to 42 units (see the horizontal dashed line in Fig 3b1).

In Fig 3a2–3b2 we plotted two traces from the first two largest clusters of the 12 shown in Fig 3b1. There are clear differences between the traces, e.g. the largest cluster in Fig 3b1 collects all LFP with small amplitude and high frequency oscillations (Fig 3a2), whereas the second largest cluster collects large amplitude LFPs that show a graded, almost linear, amplitude decay over time (Fig 3b2).

#### Dendrogram-based quantitative test

We also used the dendrogram-based clustering for measuring the percentage overlap between baseline and DA receptor antagonists trials. For each animal, we concatenated the 100 baseline trials (trial index from 1 to 100) with the 100 trials after DA receptor antagonist treatment (trial index 101 to 200) in a single 200-trial file. If the neural activity patterns for baseline and DA receptor antagonists were totally distinct, then we would expect that they separate in disjunct clusters. As a result, a perfect dendrogram classifier would correctly separates the 200 mixed baseline and dopamine antagonists trials in disjoints (non-overlapping) clusters such that each of them only contains one type of trials, either only baseline or only DA receptor antagonist trials. On the other hand, if half of the trials in a given cluster are from baseline and the other half are from DA receptor antagonists then the overlap is maximum possible and the Euclidian distance-based dendrogram cannot discriminate between those trials.

**Fig 4:**
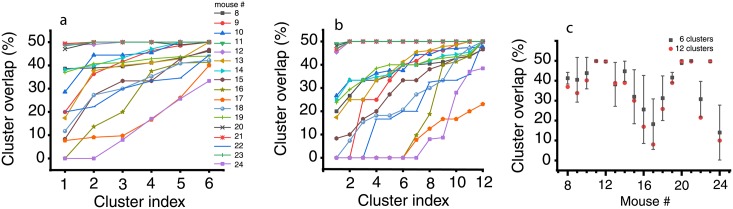
Percentage overlap between baseline and DA receptor antagonist trials. The baseline (*bc*) trials were concatenated with the corresponding DA receptor antagonist trials and classified in either 6 (a) or 12 (b) clusters. Overlapping between *bc* and DA receptor antagonists trials could be due to the limited number (six) of allowed clusters (a). Some overlapping was removed by allowing the trials to separate in 12 clusters (b). In almost all cases, the mean percentage mixing between *bc* and DA receptor antagonists trials in the same cluster is within a standard deviation regardless of the number of clusters used by classifier (c). A minimum of 20% overlap between *bc* and DA receptor antagonists suggests a possible common, invariant, part of the mathematical model that applies to both conditions. The minimum number of clusters that produce a consistent separation of trials may indicate the required number of parameters in a mathematical model that capture trials’ details.

For each cluster, we computed the percentage of mixing between baseline and DA receptor antagonists trials by using the formula [102, 103]:

$$\begin{array}{c} \%overlap=\frac{min(\#trials_{baseline},\#trials_{dopamine})}{\#trials_{cluster}}, \end{array}$$ (3)

where #*trials*_*cluster*_ represents the total number of trials in a given cluster, of which #*trials*_*baseline*_ belongs to the baseline, i.e. trial index 1 to 100, and #*trials*_*dopamine*_ belongs to the DA receptor antagonist trials, i.e. index 101 to 200. We imposed on dendrogram classifier both the option of using six (see Fig 4a) and 12 clusters (see Fig 4b) to classify the 200 baseline and DA receptor antagonists combined trials. The reason for trials classification with a different number of clusters is that a too low number of clusters would inevitably force some trials to be miss-classified. An extreme case is a dendrogram with only one cluster, which places all 200 trials together and produces a maximum possible overlap with 50% of data from baseline and 50% from DA receptor antagonists. Another extreme case is a dendrogram with 200 clusters such that each trial is place in just one cluster without any overlap. We expect that for any other arbitrarily selected number of clusters for the dendrogram classifier the overlap between the baseline and DA receptor antagonists is between zero and 50%. Although arbitrary, a reasonably small number of clusters is about six and 12 is already a too fine separation among trials to be of practical use in data modeling. The difference in the average percentage of trials overlap between 6 (solid black squares) and 12 (solid red circles) clusters is shown in Fig 4c for all mice. First, we notice that the mean percentage overlap for 6 and 12 clusters are within one standard deviation of each other in all cases. Since the average percentage overlap did not change significantly by doubling the dendrogram’s number of clusters it means that the observed overlap could be the result of true similarities among the LFP recordings as opposed to trials being forced to mix due to a too low number of available clusters for classification. Second, there are significant differences between mice (see Fig 4c) regarding the percentage mixing of baseline and DA receptor antagonist trials. For example, for mice # 16, 17, and 24 the mean overlap is less than 20%, whereas for mice 11, 12, 20, 21, and 23 the trial mixing is maximum (50%) possible.

On the low side, the 20% average mix between trials could indicate the lower bound of the correlation-based dendrogram sensitivity, which classifies some trials as “similar”, regardless how small is the correlation among them. The fact that even after circularly shifting the data we still find 20% misclassification between baseline and DA antagonist trials is an indication that the correlation-based clustering method is not perfect. The 50% average mix suggests that for the respective mice (see Fig 4c) the correlation-based dendrogram classifier is not effective in distinguishing between the baseline and DA receptor antagonist. For this purpose, we further investigated the statistics of lag times using the Fréchet distance among trials to sort out true similarities (see below).

## Lag time statistics

**Fig 5:**
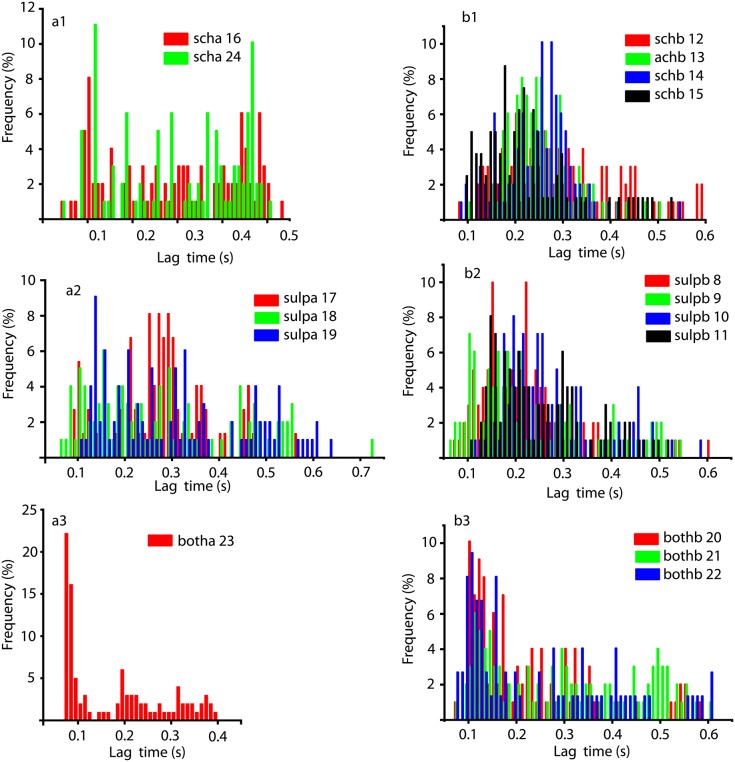
Correlation-based lag time statistics for all DA receptor antagonist conditions and mice. For *scha* condition (a1) the correlation-based lag times have a bimodal distribution with some trials having a very short correlation time around 0.1 s and another group with 0.45 s. Although this and all the other distributions are similar to their corresponding baselines (see Fig 6), they also have significant differences presumably due to the DA receptor antagonist effect. For example, *scha* condition (a1) significantly shifted towards longer lag times compared to baseline. The weighted average of all lag times were 0.3012 s for *scha* (panel a1), and 0.2072 s for the baseline (see Fig 6a1), which represents a significant 45% increase in correlation time. Similarly, the weighted average of lag times for *sulpa* was 0.2864 s (panel a2) compared to 0.2275 s for the baseline (see Fig 7), which is a 26% increase in the correlation time. For *botha* (panel a3) there was only one viable animal which showed no change in the weighted average lag times compared to the baseline (see Fig 6a3). The weighted averages of lag times were 0.2594 s for the *schb* (panel b1), 0.2439 s for *sulpb* (panel b2), and 0.2548 s for *bothb* (panel b3). In all cased when DA receptor antagonist was applied before cocaine the weighted average of all lag times is the same as the corresponding baseline value.

The autocorrelation-based lag time distributions for all six conditions listed in Table 1 are shown in Fig 5. All weighted averages of lag times were presented with four significant figures because the sampling rate was 10 kHz, i.e. a sampling time of 0.0001 s.

**Fig 6:**
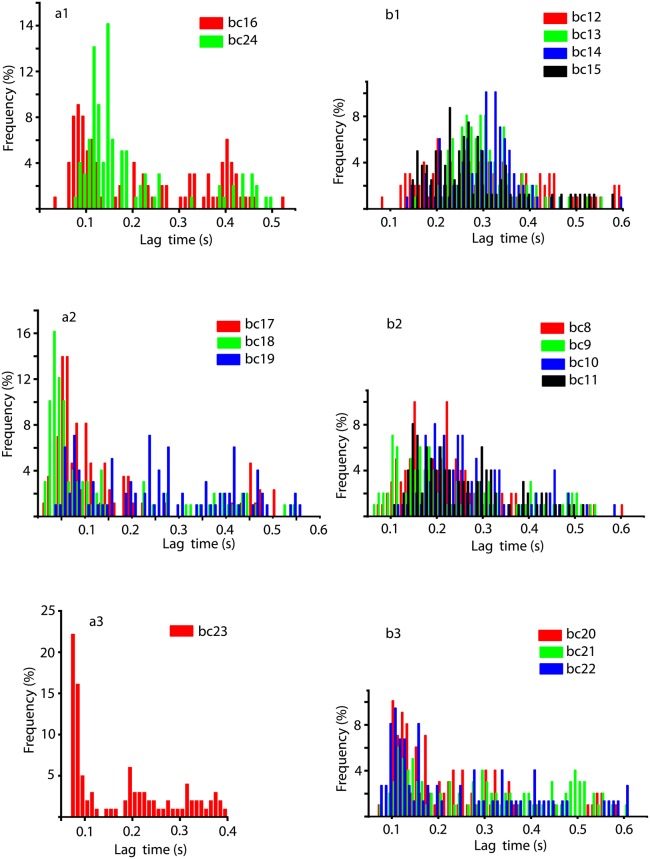
Distributions of all baseline lag times. The animal were grouped according to the DA receptor antagonist treatments shown in Fig 5 for direct comparison. Most of the distributions are unimodal, except a1 and a2. The features of the lag time distributions of the baseline were preserved both under DA receptor antagonist (see Fig 5) and under cocaine alone (see Fig 7).

**SCH 23390 after cocaine** (Fig 5a1) shows a clear bimodal distribution of lag times with one distinct peak at very low lag times (around 0.1s) and another peak at much longer lag times (about 0.45 s). For every pharmacological manipulation, such as *sch*, *sulp*, or *both* from Table 1, there is always a baseline recording for the respective mouse [102]. Such baseline recordings were called *bcxx* where *xx* is the mouse number and the corresponding distributions of lag times are shown in (Fig 6). In order to quantify the effect of DA receptor antagonists on cocaine administration, we could directly compare the distributions of lag times against the baseline distributions. For example, Fig 5a1 (SCH 23390 condition mice # 16 and 24) directly compares against Fig 6a1 (baseline recordings mice # 16 and 24). While the distribution of lag times under *scha* treatment, i.e. cocaine after SCH 23390, preserves the original bimodal distribution of the baseline it has a noticeable drift towards longer lag times. This means that the correlation time of the *scha* data increased compared to baseline.

**Fig 7:**
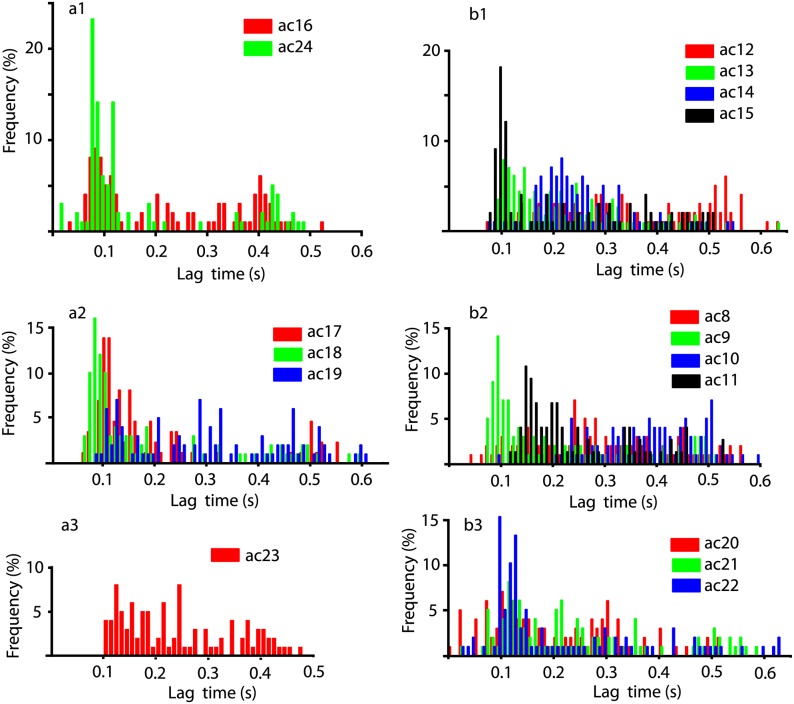
Lag time distributions under cocaine alone. While cocaine preserves the general structure of the lag time distributions noticed in the baseline (Fig 6), there are subtle differences that could be captured, for example, by the weighted average of the lag times. For example, after cocaine the weighted lag time is 0.1937 s (panel a1) and the baseline is at 0.2072 s (7% shorted compared to baseline). For panel b1 the weighed average lag time is 0.2635 s compared to 0.2594 s for baseline (2% longer than baseline). For panel a2 the weighed average lag time is 0.2275 s, which is the same as the baseline. For panel b2 the weighed average lag time is 0.2850 s under cocaine and 0.2439 s for the baseline (17% increase compared to baseline). For panel a3 the weighed average lag time is 0.2423 s after cocaine and 0.1790 s for the baseline (35% increase). For b3 the weighed average lag time is 0.2194 s for cocaine and 0.2548 s for baseline (14% shorter than baseline).

Another direct comparison is with the cocaine data [103] shown in Fig 7. As we previously reported, cocaine generally shifts the lag times towards shorter durations compared to baseline values [103]. If we compare the peak around the lag time of 0.1 s in the baseline Fig 6a1 against the cocaine Fig 7a1, then not only that the peak narrowed under cocaine but it is also much stronger compared to any other lag times. More quantitatively, the weighted average of all lag times for the baseline was 0.2072 s, 0.1937 s after cocaine, and 0.3012 s after *scha* (see Table 3). In this study, we expanded our previous analysis [102, 103] from six to 17 animals and noticed that sometimes the cocaine increases the weighted average of lag times compared to baseline. However, to put this apparently contradictory result in statistical perspective, we notice that the largest increase of 35% is only observed in one animal, i.e. *bc*23. The other increase of 17% compared to the baseline is also due to only one animal *bc*10 that skews the entire average of the group. In all the other 15 animal the cocaine shifts the weighted average lag times to shorter durations compared to the baseline as previously reported in [103].

**Table 3:** Autocorrelation-based lag time averages (in milliseconds) for baseline, DA receptor antagonists before cocaine, cocaine, and DA receptor antagonists after cocaine. The first column represents the baseline recording, the second column represents data from DA receptor antagonists applied before cocaine, the third column represents data collected when cocaine alone was applied, and the last column are data recorded when DA receptor antagonists was applied after cocaine. The respective DA receptor antagonists are listed in parentheses. All standards deviations are given with two significant figures.

Baseline	DA antagonist before cocaine	Cocaine	DA antagonist after cocaince
259 ± 92	259 ± 92 (sch)	260 ± 120	
240 ± 100	240 ± 100 (sulp)	290 ± 200	
250 ± 140	250 ± 140 (both)	220 ± 140	
210 ± 130		190 ± 140	300 ± 140 (sch)
230 ± 140		230 ± 140	270 ± 130 (sulp)
180 ± 110		240 ± 110	180 ± 110 (both)

This confirms the observation that generally cocaine alone shifts the lag times towards smaller durations compared to baseline [103], i.e. decreases the correlation time of the data, whereas SCH 23390 applied after cocaine significantly shifts the lag times towards longer durations. In this study, we used the weighted average of the lag times to put a number on a complex and complicated distribution of lag times. In our previous studies, we fitted the lag time distributions with log-normal functions and extracting the peak of the fitting function (see [103]). The main reason for such a change in our approach is that a log-normal fit, while appropriate for the six mice considered in [103], it is not appropriate for all distributions in this 17 mice study. Indeed, some distributions are clearly bimodal, such as the one shown in Fig 5a1, which would be a very poor log-normal fit.

**SCH 23390 before cocaine** lag time distributions (Fig 5b1) are unimodal with a weighted average of lag times around 0.26 s. The range of lag times is similar for both SCH 23390 conditions and is shorter than 0.6 s. The unimodal shape reflects the original baseline unimodal shape of the lag time distribution in Fig 6b1. The weighted average of lag times for baseline was 0.2594 s (see the first column in Table 3), which shifted to 0.2635 s under cocaine (see Fig 7b1), which remained unchanged when DA receptor antagonists were applied before cocaine (see the second column in Table 3). The shift in the lag time when cocaine follows the DA receptor antagonists is shown in the third column of Table 3. When SCH 23390 was applied before cocaine (second column in Table 3) it facilitated the shift of the lag times back towards the baseline values (first column in Table 3) and canceled out the effect observed in cocaine trials (third column in Table 3). When SCH 23390 was applied after cocaine (fourth column in Table 3) it facilitated the occurrence of longer lag times compared to baseline.

**Sulpiride after cocaine** (Fig 5a2) also shows a bimodal distribution with one peak around 0.25 s and the other, significant but less prominent, around 0.5 s. The bimodal distribution is more prominent in the baseline histograms shown in Fig 6a2 and also somewhat preserved under cocaine (see Fig 7a2). The weighted average of lag times for the baseline was 0.2275 s, remained unchanged under cocaine (third column in Table 3), and then shifted to a longer duration of 0.2864 s when sulpiride was applied after cocaine (the fourth column in Table 3). We noticed that when either DA receptor antagonists sulpiride or SCH 23390 are applied after cocaine (see the fourth column in Table 3), they increase the lag time compared to baseline (see the first column in Table 3).

**Sulpiride before cocaine** trials (Fig 5b2) have almost unimodal distributions of lag times with a peak around 0.2 s. The range of both distributions is a bit over 0.6 s. The weighted average of lag times was 0.2439 s for the baseline (Fig 6b2), shifted to 0.2850 s under cocaine (Fig 7b2), and returned to the baseline value of 0.2439 s when sulpiride was applied before cocaine (Fig 5b2).

**Both SCH 23390 & Sulpiride after cocaine** is unimodal (Fig 5a3), although we only retained one good data set. The weighted average of lag times for the baseline was 0.1790 s (see Fig 6a3), 0.2423 s after cocaine (see Fig 7a3), and 0.1790 s for both sulpiride and SCH 23390 after cocaine (see Fig 5a3).

**Both SCH 23390 & Sulpiride before cocaine** clearly show unimodal lag time distributions (see Fig 5b3). As opposed to SCH 23390 or sulpiride alone, the lag times in this case concentrate around very short values (around 0.12 s) and the distribution has a long tail of significant contributions up to 0.6 s. The weighted average of lag times was 0.2548 s for baseline (see Fig 6b3), 0.2194 s for cocaine (see Fig 7b3), and 0.2548 s when sulpiride and SCH 23390 were applied together before cocaine (see Fig 5a3).

To summarize, regardless of the effect of cocaine when applied alone (the third column in Table 3), the combination DA receptor antagonists followed by cocaine (the second column of Table 3) always leaves the lag time at the baseline values. On the other hand, when DA receptor antagonists is applied after cocaine (the fourth column in Table 3) it increases the lag time compared to the baseline. There is one exception to this observation (see the last line in Table 3), but it could be an outlier since there was only one set of data available for this particular condition.

**Fig 8:**
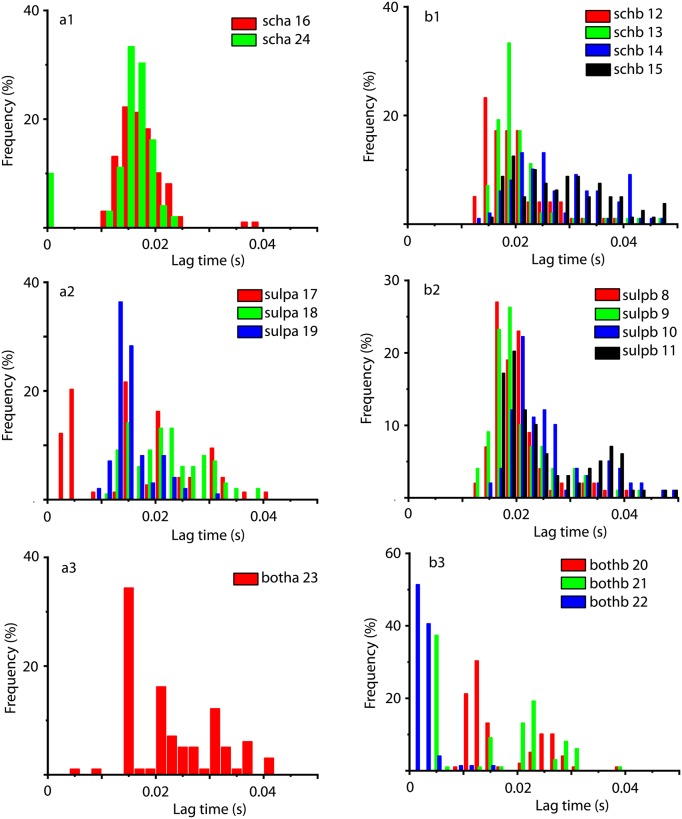
Average mutual information-based lag time statistics for all DA receptor antagonist conditions and mice. For *scha* condition (a1), the AMI-based lag times have narrow unimodal distributions with an average around 0.017 s (see Table 4 for mean ± standard deviation values). For *schb* condition (a2), the AMI-based lag times is unimodal with an average of 0.024 s, although for mice 14 and 15 the distributions have long tails that extend past 0.04 s. For *sulpa* condition (b1), the AMI-based lag times have unimodal distributions with an overall average of about 0.017 s. For *sulpb* condition (b2), the AMI-based lag times have unimodal distributions with an overall average of about 0.23 s and long tails that extent past 0.04 s. For *botha* condition (c1), the AMI-based lag time distribution has and average of about 0.023 s and a long tail that extent past 0.04 s. For *bothb* condition (c1), the AMI-based lag time distribution has and average of about 0.012 s and a long tail up to 0.04 s.

Below we briefly summarize the results on the lag times obtained from AMI in Fig 8 and Table 4. When comparing the lag time obtained with the autocorrelation method (see Fig 5) against AMI lag times (see Fig 8) one obvious difference is that AMI lag times are about an order of magnitude shorter.

**Table 4:** Average mutual information-based lag time averages (in milliseconds) for baseline, DA receptor antagonists before cocaine, cocaine, and DA receptor antagonists after cocaine. The first column represents the baseline recording, the second column represents data from DA receptor antagonists applied before cocaine, the third column represents data collected when cocaine was applied, and the last column are data recorded when DA receptor antagonists was applied after cocaine. The respective DA receptor antagonists are listed in parentheses. All standards deviations are given with two significant figures.

Baseline	DA antagonist before cocaine	Cocaine	DA antagonist after cocaince
24.1 ± 7.0	24.1 ± 7.0 (sch)	24.8 ± 7.3	
23.1 ± 6.7	23.1 ± 6.7 (sulp)	23.4 ± 7.0	
12.0 ± 6.2	12.0 ± 6.2 (both)	7.7 ± 3.7	
21.3 ± 5.8		20.6 ± 5.5	17.0 ± 3.4 (sch)
16.8 ± 5.5		16.8 ± 5.5	17.8 ± 6.8 (sulp)
22.8 ± 8.1		17.4 ± 5.6	22.8 ± 8.1 (both)

One benefit of the AMI-based lag times analysis is the identification of a few faulty couplings between the optrode and the tissue. Lag times of the order of 1-5 ms could be realistic when studying small neural networks, since they are of the same order of magnitude as the durations of individual action potentials. However, such short lags are unrealistic when measuring LFPs that average processes over hundreds and possibly thousands of individual action potentials. We noticed a few unusually short lag times (between 2 ms and 5 ms) while most of the lag times are above 0.01 s. As shown in Fig 8, such examples are mouse 17 (about 30% of the lag times below 5 ms), mouse 22 (over 85% of the lag times below 5 ms), and mouse 21 (over 40% of lag times below 5 ms). A closer inspection of all these particular trials showed that the LFPs have very low peak-to-peak amplitude (below 0.04) and they were covered by noise, probably due to faulty coupling with the optrode.

One immediate conclusion from comparing autocorrelation-based lag times from Table 3 against the AMI-based lag times from Table 4 is that the standard deviation of AMI-based lag times is smaller in most cases. This suggests a narrower distribution of lag times for AMI-based method. Another conclusion is that the correlation-based lag time (see Fig 5) is larger than the AMI-based lag time (see Fig 8) by an order of magnitude. With such a wide spread of lag times, the concern is that the attractors may not unfold properly. We found that attractors unfold both with the AMI-based and autocorrelation-based lag times.

### Kolmogorov-Smirnov (KS) test

We are both interested in determining the exact values of the delay (or lag) time and also check if the lag time distributions differ from one condition to another. This is the second statistical test, in addition to the previously performed dendrogram clustering of similar trials, aimed at identifying statistically significant variability among trials for the purpose of subsequent mathematical modeling. We are interested in checking if baseline trials which previously could not be distinguished from DA receptor antagonist trials using dendrogram classifiers are in any way distinct from each other with respect to lag time distributions.

Although the plots of the distributions in Figs 5–7 offer a good qualitative comparison among them and the wighted average of lag times (see Table 3) gave a quantitative description of the expected lag, we also performed a direct comparison of lag time distributions. Multiple conventional statistical significance test methods can be used, such as two-tailed t-test, two-sample Kolmogorov-Smirnov test, Wilcoxon rank sum test, analysis of variance, and Kruskal-Wallis test.

**Fig 9:**
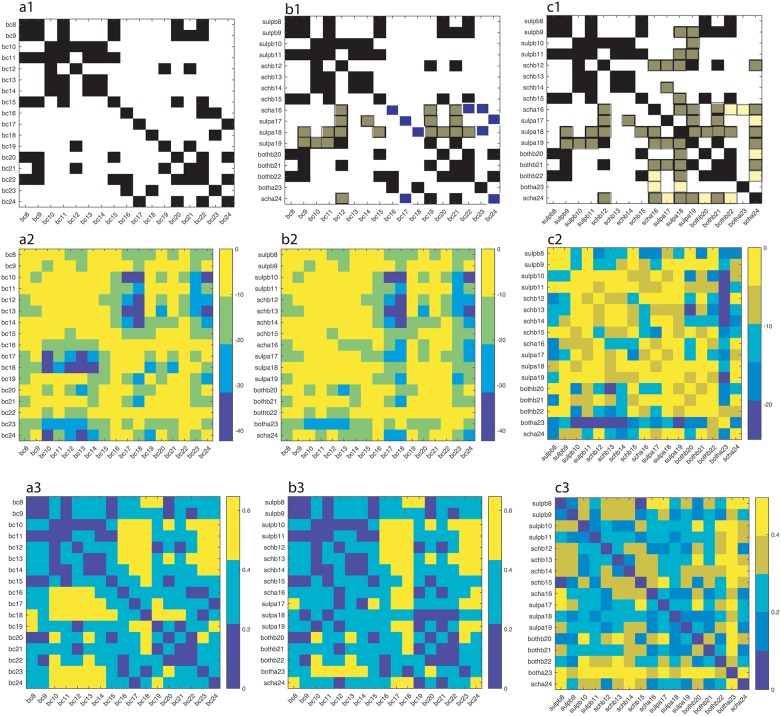
Kolmogorov-Smirnov tests. Solid black squares indicate lag time distributions belonging to the same class (a1). For example, *bc*8 and *bc*9, *bc*11, *bc*15, *bc*20 and *bc*22 are likely to be drawn from the same distribution. The measure of likelihood is determined by p-value, which is given on a natural logarithmic scale in panel (a2) where the bright orange color covers the range from *e*^0^ = 1 to *e*^−10^ ≈ 4.5 × 10^−5^. The maximum difference between any two distributions is shown in panel (a3). Dark colors mean small differences between empirical distributions. Panels a1-a3 refer to baseline distributions and are only provided to help us understand the changes induced by DA receptor antagonists (panels b1-b3). Panel b1 is similar to a1 (baseline), except for a few conditions and mice that seem to be affected by DA receptor antagonists. For example, the mouse # 18 only had similar distribution of lag times with mouse #23 under baseline (see panel a1), whereas when treated with *sulpa* (sulphiride after cocaine) its lag time distribution became similar to baseline lag time distributions for *bc*9, *bc*11, *bc*12, *bc*15, *bc*19, *bc*20, *bc*21, and *bc*22. These new correlations are shown with solid red squares to signal that they are new and unexpected compared to baseline. At the same time, *sulpa*18 distribution lost its baseline similarity with *bc*18 and *bc*23, which is marked with solid blue triangles in panel b1. The corresponding p-values (panel b2) confirm the new membership of the lag time distributions to the set *bc*9, *bc*11, *bc*12, *bc*15, *bc*19, *bc*20, *bc*21, and *bc*22. Furthermore, the maximum distance between *sulpa*18 and *bc*9, *bc*11, *bc*19, *bc*20, *bc*21, and *bc*22 is indeed low (below 0.2), whereas it increases a bit when compared to *bc*12 and *bc*15, but remains below 0.4 (panel b3). When cross-correlating the DA receptor antagonists’ effects against themselves (a3) we notice a significant increase similar lag time distributions (solid red squares) compared to the baseline (solid black squares). Some previously similar distributions in the baseline, e.g. *bc*16, *bc*22, and *bc*23, are lost under DA receptor antagonists (marked with solid green squares).

Kolmogorov-Smirnov test [145] was used here for deciding if a sample comes from a population with a specific distribution. If the data come from the same distribution at the default 5% significance level, then we represented that result with a solid black square in Fig 9a1–9c1. The probability of observing a test statistic as extreme as, or more extreme than, the observed value under the null hypothesis is shown on a natural logarithmic scale in Fig 9a2–9c2. Statistical significance is often referred to as the probability value, or p-value. A small p-value means that the data are unlikely under some null hypothesis. Usually, the null hypothesis is rejected if *p* < 0.05. We also computed the maximum difference between empirical distribution functions and plotted them in Fig 9a3–9c3.

To understand how much of the pharmacological manipulation changes the distributions of lag times, we first run the KS test on the baseline data (Fig 9a1). Any off-diagonal black square indicates that the respective baseline distributions are from the same empirical distribution. For example, the lag times for *bc*8, *bc*9, *bc*11, *bc*15, *bc*20, and *bc*22 seem to all follow the same distribution.

The next comparison is among the distributions of the baseline versus DA receptor antagonists trials (see Fig 9b1). For example, the mouse #8 has been treated with sulpiride before cocaine (*sulpb*) and the distribution of delay times for *suplb*8 is similar to *bc*8, *bc*9, *bc*11, *bc*15, *bc*20, and *bc*22. This means that for this mouse the sulpiride injection did not change the lag time distribution and, therefore, the black squares remained in the same place as in the baseline case shown in Fig 9a1.

For other mice, such as #18 it turned out that the injection of sulpiride after the cocaine (*sulpa*) changed the distribution of lag times and made it more similar to *bc*9, *bc*11, *bc*12, *bc*15, *bc*19, *bc*20, *bc*21, and *bc*22 (see the solid red squares in Fig 9b1). At the same time, for mouse # 18 the *sulpa* distribution is definitely different than its baseline (*bc*18) and also different from *bc*23 (see the solid blue squares in Fig 9b1). Notice that in the baseline case, *bc*18 and *bc*23 lag time distributions are of the same type. This suggests that the combination cocaine followed by sulpiride is affecting the dynamic reconstruction of the attractor through the correlation lag time of data.

When compared against the baseline lag time distributions, the changes seem to occur in *scha*16, *sulpa*17, *sulpa*18, *sulpa*19, and *scha*24 recordings (solid red squares in Fig 9b1).

Finally, when computing the KS test across all DA receptor antagonist trials (see Fig 9c1) we notice an increase in the fraction of similar distributions compared to the baseline. For example, mouse #24 had similar distributions with mice # 17, 20, and 22 (see solid black squares in Fig 9a1). However, *scha* treatment made the lag time distributions for mouse #24 similar to those of mice # 12, 16, 18, 19, and 21 (see the solid red squares in Fig 9c1). Notice that before treatment the distribution of lag times for #24 was different from the baseline for # 12, 16, 18, 19, and 21. We also noticed that *scha* treatment removed similarities with the baseline # 17, 20, and 22, which is shown with green solid squares in Fig 9c1.

## Reconstructed neural activity under DA receptor antagonists

### The lag time

The distribution of all autocorrelation-based lag times are shown in Fig 5 and the AMI-based lag time values (not shown) were within 5% of those obtained with the autocorrelation method. As before [102, 103], we used the Tisean function *autocor* to compute the autocorrelation and *mutual* to compute the AMI.

### The embedding dimension

The lag time was used for computing the embedding dimension of the data over distance ratios, *f*, between 2 and 20 and Theiler windows from 100 to 8000 sampling times (see [102, 103] for explicit Tisean function calls). We found that the percentage of FNN dropped below 0.1% for an embedding dimension of *d*_*E*_ = 3 and *f* > 10.

**Fig 10:**
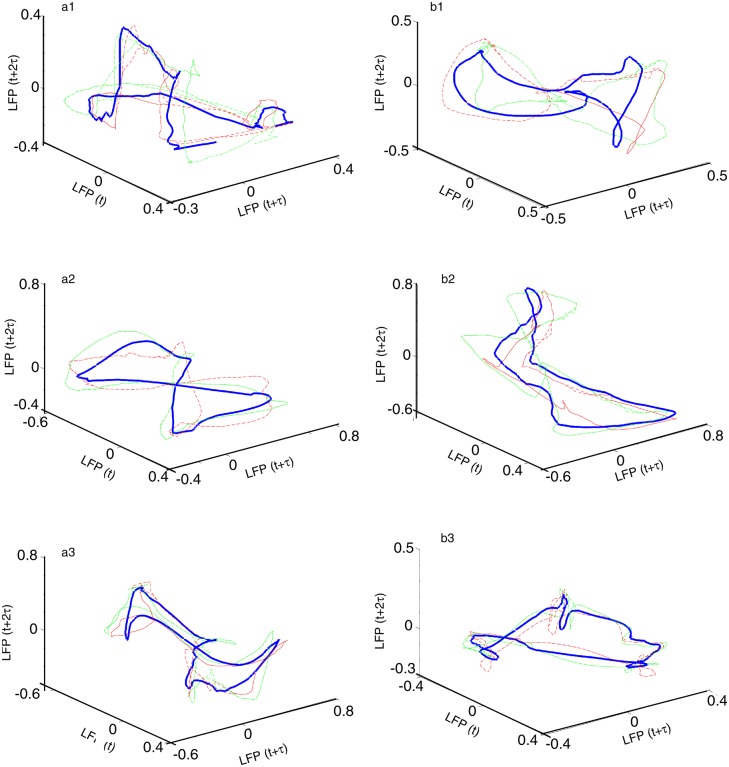
Three-dimensional reconstructed attractors of neural activity. Representative reconstructed attractors of neural activity under each condition: *scha* (a1), *schb* (b1), *sulpa* (a2), *sulpb* (b2), *botha* (a3), and *bothb* (b2). For each condition only two traces are shown with thin continuous and dashed lines, respectively. The thick line is the average reconstructed trace over the entire cluster, which is provided only as a visual aid. The trajectories resemble those observed under baseline [102] and cocaine [103].

The phase space attractor of each trial was obtained but we only show two representative examples from each dendrogram-based cluster (see Fig 10). Although the attractors for different clusters look different, they are topologically equivalent (see [102, 103]), i.e. by changing the delay time one phase space trajectory could be morphed into the other. The attractors in Fig 10a1 (*scha*), a2 (*sulpa*), and a3 (*botha*) show an “8”-shape, which is topologically equivalent with a closed elliptic attractor as shown in Fig 10b1 (*schb*), b2 (*sulpb*), and b3 (*bothb*). Indeed, the attractor in Fig 10a1–10a3 could be morphed into the one shown in Fig 10b1–10b3 by twisting the upper half of the attractor with respect to the lower half.

**Fig 11:**
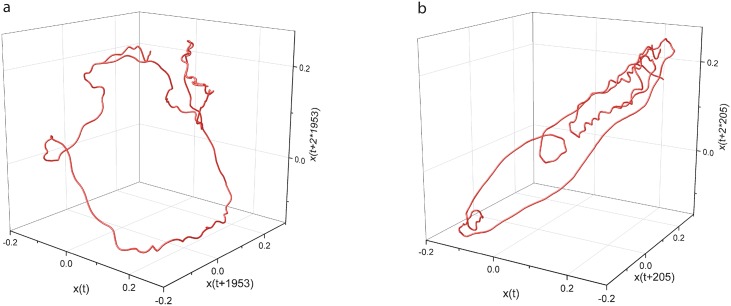
Three-dimensional reconstructed attractors both with autocorrelation-based and AMI-based lag times. Representative reconstructed attractors of neural activity from *schb*12 trial #5 with a lag time of 1953 sampling times, i.e. 195.3 ms, based on autocorrelation method (a), and with 205 lag times, i.e. 20.5 ms, based on AMI method (b). While the lag times are one order of magnitude apart, they still unfold the attractor without any self-crossings of the phase space trajectory (see also the three-dimensional rotating frame in S1 Fig (for correlation-based lag time reconstruction) and S2 Fig (for AMI-based lag time reconstruction)).

As mentioned in the lag time section, the autocorrelation-based lag time (see Table 3) was an order of magnitude larger than the AMI-based lag time (see Table 4). This raised the question whether such low lag times could properly unfold the three dimensional attractor as we observed for much larger autocorrelation-based lag times in Fig 10. We show below a side-by-side example of an attractor for the same condition and the same trial unfolded both with the autocorrelation-based and AMI-based lag time in Fig 11. For the example shown in Fig 11, the condition, the mouse, and the trial were randomly selected from among the six conditions, 24 mice, and 100 trials for each mouse.

### Fréchet distance in neural activity space

**Fig 12:**
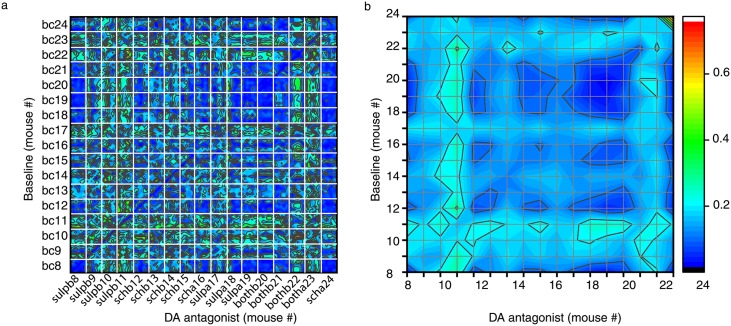
Fréchet distance between baseline and DA receptor antagonists. (a) Each white-bordered square contains the color-coded Fréchet distance between the 100 control (*bc*) trials and the 100 DA receptor antagonist trials. Deep blue colors mean small Fréchet distance and suggest similar phase space trajectories. (b) The average over each 100 × 100 block offers a smoother color-coded overview of topological similarities among conditions. For example, there are a few clusters of similar geometrical structure among the three-dimensional attractors that are below 10% of the maximum observed arbitrary Fréchet distance. Among them, *bc*8 are topologically similar to *sulpb*8, *sulpa*18, *sulpa*19, and *bothb*20. Similar clusters are *bc*19 & *bc*20 and *sulpb*19 & *sulpb*20 and a larger blue cluster with *sulpa*18, *sulpa*19, and *bothb*20 (see upper right side of panel b).

Finally, we performed a third statistical test of similarities among trials based on the reconstructed tree-dimensional attractors. For this purpose, we computed the Fréchet distance [133, 135] between three-dimensional phase space trajectories, such as those shown in Fig 10. Intuitively, Fréchet distance relates to the “man walking a dog on a leash” paradigm, i.e. find the shortest leash needed such that the man walks along one curve and the dog walks along the other [134]. Fréchet distance is a topological measure of the similarity between two curves [134], i.e. it does not consider any temporal constrains regarding the location of figurative points on the two curves. We computed Fréchet distance using the algorithm in [136]. In Fig 12, each white-bordered square corresponds to a combination of 100 baseline trials × 100 trials with DA receptor antagonists. For example, the top left square shows the color-coded Fréchet distance between the 100 baseline trials *bc*8 and the 100 trials after DA receptor antagonist *sulpb*8.

Fréchet distance is a topological measure of similarity between two curves, i.e. once the lag time was used for the purpose of unfolding the attractor the structure is “frozen” in phase space. This means that Fréchet distance only computes the topological distance between any two points of the two trajectories regardless of the speed at which the figurative points travel along the path, i.e. disregards dynamical aspects.

The fact that two different conditions, such as *bc*8 and *sulpb*8 have similar-looking three-dimensional attractors at an average minimum distance of 0.03 ± 0.01 arbitrary units (indicated by blue color in Fig 12) means that the figurative points of the two conditions visit similar phase space regions of neural activity. At the same time, for the same two conditions the weighted average lag times is 0.244 s both for the baseline (Fig 6b2) and *sulpb*8. This means that the two trajectories are similar both geometrically and that they are traveled at the same speed. Other conditions, such as *bc*9 and *sulpb*9, have quite large Fréchet distance of 0.15 ± 0.02 between reconstructed attractors despite the fact that their lag time distributions have the same weighted average lag times. The implication is that although the time constants for the phase space dynamics are identical for mice # 8 and 9 both for the baseline and DA receptor antagonist, the differences in topological distances suggest that either the initial conditions for the two mice were different or the sensitivity to light stimuli for the two mice is systematically different.

### Towards a quantitative model of the experimental data

**Fig 13:**
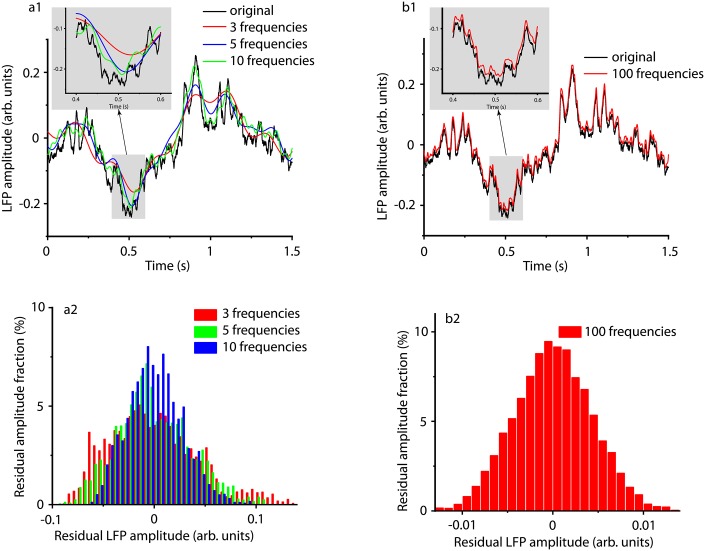
Synthesized traces from the strongest harmonics of the original signal. The original LFP recording (black) was first decomposed in Fourier harmonics and then a signal was synthesized from the first 3 (a1 red line), 5 (a1 blue curve), 10 (a1 green curve), and 100 (b1 red curve) frequencies with the largest amplitude in the power spectrum. The insets show that as we include more harmonics, the synthesized signal better approximates the original LFP recording. All distributions of residuals, i.e. the difference between the original and the synthesized signal, have Gaussian shapes (a2 and b2). The amplitude or residuals decreases by an order of magnitude as the number of frequencies for the synthesized signal increased from 3 (a2) to 100 (b2).

The reconstructed attractors (see Figs 10 and 11) suggest closed phase space, i.e. periodic, trajectories. While in principle any signal could be represented as an infinite series of harmonics, we only decomposed the signals using 3, 5, 10, and 100 of the harmonics with the highest amplitudes (see Fig 13). As the inset in Fig 13a1 shows, including more frequencies in the spectral analysis leads to a better approximation of the original LFP. The distribution of residuals, i.e. the difference between the LFP and the synthesized signal, shown in Fig 13a2 suggests that the spread of the distribution decreases as the number of harmonics increases. When 100 frequencies with the highest amplitude are considered (Fig 13b1), then the synthesized signal can reproduce minute details of the LFP and the corresponding residual has a Gaussian distribution with a very narrow range (see Fig 13b2).

We tested the LFP residuals obtained by subtracting the synthesized signal according to Eq 2 from the original LFP data (see Fig 13a2 and 13b2). In all signals synthesized with more than 10 frequencies the residuals were identified with Matlab system identification toolbox as ARMA(0,0) processes, i.e white noise. Additionally, we ran the augmented Dickey-Fuller and Kwiatkowski, Phillips, Schmidt, and Shin (KPSS) tests. Both tests showed that the residual time series shown in Fig 13 were stationary. When we used less than 10 frequencies of the Fourier spectrum to synthesized the signal, the residual signal failed one or both stationarity tests. This shows that with a too low number of harmonics the residual signal still contains trends that were not removed from the original signal when subtracting the synthesized one.

**Fig 14:**
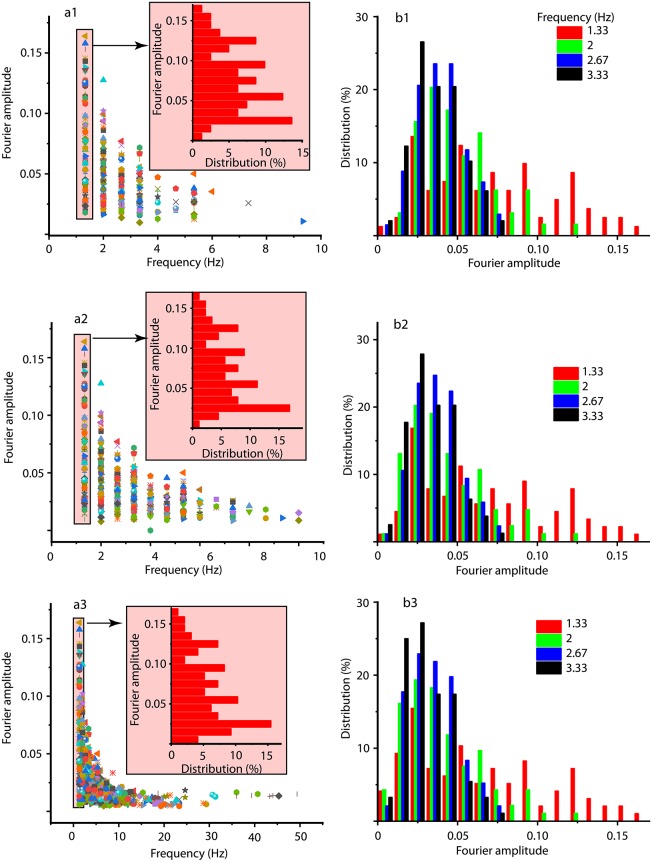
Fourier amplitudes versus frequency for signal synthesis. The Fourier amplitudes for the first 3 (a1), 5 (a2), and 10 (a3) strongest harmonics over all trials of *sulpa* for mouse #18. Each frequency has an amplitude range (see shade narrow rectangle) that can be better capture by its distribution (see the corresponding shaded inset). The insets in panels (a) show the percentage contribution (horizontal axis) of each Fourier amplitude (vertical axis) for a particular frequency. Panels (b) show the distribution of Fourier amplitudes of the first four strongest harmonics.

The summary of spectral analysis over all trials of *sulpa* for mouse #18 is shown in Fig 14a1 (first three strongest frequencies in Fourier spectrum), a2 (first five frequencies), and a3 (first ten frequencies). As Fig 14a1–14a3 suggest, for each frequency in the Fourier spectrum (horizontal axis) the corresponding amplitude has a consistent distribution (see the insets) regardless the number of selected frequencies. Fig 14b1–14b3 summarize the distribution of Fourier amplitudes over all trials of *sulpa* for mouse #18 for the first four frequencies.

We present in the Supporting Information the extensive S1 Table that contains all the frequencies and Fourier amplitudes necessary for the synthesis of the signal with three, five, and 10 highest amplitudes. We did not include in the table the model with 100 highest amplitudes since it only adds extra coefficients with little extra information compared to the already presented reduced models. First, we noticed that different trials may have different spectral decompositions. For example, when decomposed in three Fourier components, trial #1 requires frequencies *k* = 2, 4, 6 whereas trial # 15 requires frequencies *k* = 2, 4, 8. As a result, for each of the three decompositions in Fourier components, we also show what fraction of the trials required a particular frequency. Based on the number of data points and the sampling duration, the actual frequency is *f*_*k*_ = *k*/3 (Hz). Since in Fig 14 we only showed the distribution of amplitudes for the first four strongest frequencies, we show below a very small excerpt from S1 Table.

We notice from Table 5 that the first four frequencies have stable amplitudes over different Fourier representations of the signal, which is an indication that they capture a significant amount of information regarding the LFPs. Even in the case of a flexible decomposition of LFPs with 10 frequencies, the four strongest harmonics listed cover over 44% of the trials and 61% of the trials when constraining the Fourier decomposition to five frequencies (see S1 Table for detailed information on Fourier coefficients and their corresponding weights in data modeling).

**Table 5:** Fourier coefficients for the first four strongest frequencies in Fourier spectrum. The Fourier amplitudes for *sulpa* mouse #18 shown in Fig 14 for the 100 trials with three Fourier coefficients (the second and third columns), five coefficients (the fourth and fifth columns), and 10 coefficients (the sixth and seventh columns). The first column represents the integer frequency index *k*. Although each trial was synthesized using the largest 3, 5, or 10 Fourier amplitudes, different trials have different combinations of frequencies. For each of the three decompositions in Fourier components, we show what fraction of the trials required a particular frequency.

k	% trials (3 coeff)	*A*_*k*_ (3 coeff.)	% trials (5 coeff.)	*A*_*k*_ (5 coeff.)	% trials (10 coeff.)	*A*_*k*_ (10 coeff.)
4	31	0.059 ± 0.038	23	0.059 ± 0.041	12.9	0.059 ± 0.037
6	20	0.091 ± 0.068	16	0.083 ± 0.069	9.1	0.083 ± 0.067
8	15	0.125 ± 0.090	11	0.125 ± 0.097	6.1	0.125 ± 0.090
10	15	0.125 ± 0.092	11	0.125 ± 0.10	6.1	0.125 ± 0.10

To conclude, a spectral model of the data with less than ten harmonics is accurate enough. Given the large number of trials that are captured correctly even by only four harmonics (see Table 5), a much lower number of Fourier coefficients (maybe five) may suffice.

## Discussion

What did we learn from analyzing the neural activity under DA receptor antagonists? First, we found that the delay embedding method works (see [102] for a discussion of control data and [103] for cocaine data). Second, we found that the embedding dimension is still three, the same as in the previous studies [102, 103]. This means that the mathematical model that could describe the control, the cocaine, and DA receptor antagonists only require three independent variables.

We also found that there are mice for which the overlap between baseline and DA receptor antagonists is maximum, i.e. 50% and, therefore, the dendrogram with Euclidian distance measure cannot distinguish between baseline and DA receptor antagonists. Such examples are mice # 11, 12, 20, 21, and 23. We also caution against exclusively and solely relying on dendrogram for the purpose of trials’ classification and statistics. For example, the dendrogram is sensitive to the type of distance selected for classifier.

### Weighted average of lag times

The second, more qualitative classifier was based on the distribution of lag times. The delay times for the phase space reconstructions significantly differ among animals (see Fig 5). Although the optimal delay time even differs from trial to trial, it is clear that the distributions of delay times are also significantly different between baseline (Fig 6) and DA receptor antagonists for most mice (see Fig 5).

For some mice, the LFPs of baseline and DA receptor antagonists were not distinguishable using dendrogram method, whereas the lag time statistics allowed a clear separation of the two conditions. For example, for mouse # 12 the weighted average of lag times for baseline was 0.297 s which shifted to a lower lag times of 0.26 s when SCH 23390 was applied before cocaine (see Fig 7b1).

On the other hand, the weighted average of lag times for other mice did not change between baseline and DA treatments. For example, for the mouse # 11 the weighted average of lag times was 0.244 s (Fig 5b2), for mice # 20 and 21 it was 0.255 s (see Fig 6b3), and for mouse # 23 it was 0.178 s (see Fig 6a3). At the same time, only comparing DA receptor antagonists against baseline does not cover the entire complexity of the experiment we carried out. This is because the common denominator of all DA receptor antagonist experiments in this study is cocaine: we either injected cocaine **before** or **after** the D1 receptor antagonist SCH 23390, or the D2 receptor antagonist sulpiride, or both SCH 23390 & sulpiride. Therefore, we also checked DA receptor antagonists’ effect on lag time distributions when compared against pure cocaine experiments [103]. We are again interested in mice # 11, 12, 20, 21, and 23 since the dendrogram could not distinguish between baseline and DA receptor antagonist trials in these cases. As it turned out, in all of the above cases there were significant differences between the lag times of cocaine trials (see [103]) and DA receptor antagonists. For example, the weighted lag time for mouse # 11 is 0.285 s under cocaine and 0.244 s when sulpiride was applied before cocaine (Fig 5b2). The weighted lag time for mouse # 12 was 0.263 s under cocaine and 0.26 s when SCH 23390 was applied before cocaine (Fig 5b1). The weighted lag times for mice # 20 and 21 were 0.219 s for cocaine and 0.255 s for sulpiride and SCH 23390 applied before cocaine (see Fig 5a3). The weighted lag times for mouse # 23 was 0.242 s after cocaine and at 0.178 s for both sulpiride and SCH 23390 after cocaine (see Fig 5a3).

It is also important to note that when compared against baseline, the only trials in which the weighted average of the lag time increased were *scha* and *sulpa*. In all other conditions, i.e. *schb*, *sulb*, and *both*, the weighted average of the lag times remained the same. This indicates that from a dynamic point of view, it matters in which order is the SCH 23390 and sulpiride, respectively, injected with respect to cocaine. In trials where the DA receptor antagonists were injected **after cocaine the lag time increased**, which means a longer correlation time of the LFP recordings and, therefore, a different time scale of the model equations compared to the baseline. At the same time, when cocaine was applied **before** DA receptor antagonists we did not observe changes in the weighted average of the lag times compared to baseline. To fully understand these cases we need to compare them against the effect of pure cocaine (no DA receptor antagonists). We noticed that in *sulpb*, such as *sulpb*9 and *sulpb*11, the cocaine alone decreased the weighted average of the lag times compared to baseline whereas **sulpiride applied before cocaine** shifted the lag times to longer durations and precisely back to the baseline, practically **canceling the effect of cocaine**. Similar responses were observed in *schb*13 and *schb*15 where cocaine alone decreased the weighted average of the lag times compared to baseline and then **SCH 23390 applied before cocaine practically canceled the effect of cocaine** leaving the lag time at baseline values. Finally, in all cases when both SCH 23390 & sulpiride were applied before cocaine, i.e. *bothb*20, *bothb*21, and *bothb*22, the cocaine alone systematically moved the weighted average of the lag times to shorter durations compared to baseline, whereas the two DA receptor antagonists together practically canceled out the effect of cocaine and left the lag times at baseline values.

It is also important to address the issue of some trials that do not fit the above picture, such as *sulp*8 and *sulp*10 for which cocaine alone shifted the weighted average of the lag times to longer durations. Even in these two cases, the effect of sulpiride was consistent with the other *sulpb* cases, i.e. sulpiride applied before cocaine shifted the lag times back to baseline. The mismatch does not concern the effect of DA receptor antagonists, which consistently cancel out the effect of cocaine when applied before it, but rather the unusual increase in the weighted average of the lag times under cocaine. The cause could be our choice of the weighted average of the lag times as a measure for comparing two distributions, i.e. baseline and cocaine. In contrast, in [103] we first fitted the distributions with a log-normal function and then compared the location of the peaks of the distributions. In particular, *sulpb*8 and *sulpb*10 have a longer tail compared to *sulpb*9 and *sulpb*11 that shifts the weighted average of lag times towards longer durations while the peak of the log-normal distribution remains unchanged (not shown). Therefore, the mismatch could be determined by our choice of the number we put on a distribution of lag times. Furthermore, for the particular mice # 8 and 10, a log-normal fit is not quite optimal as the lag time distributions seem to have bimodal shapes.

### Kolmogorov-Smimov (KS) similarity of lag time distributions

The KS test that compared the actual distributions of baseline lag times against DA receptor antagonists (see Fig 9b1) has a significantly large number of diagonal elements, i.e. cases where KS test placed the baseline and DA receptor antagonists in the same empirical distribution. Among others, we find again mice # 11, 12, 20, 21, and 23 in this category. Interestingly, DA receptor antagonists removed mice # 16, 17, 18, and 24 from the cross-list and indicated that their lag time distributions changed compared to baseline (see blue solid squares in Fig 9b1). This confirms the findings based on the weighted average of the lag times and supports the conclusion that DA receptor antagonists applied after cocaine, i.e. *scha*16, *scha*24, *sulpa*17, *sulpa*18, increased the lag time/correlation time of LFP recordings. The two missing mice for DA receptor antagonists applied after cocaine, i.e. *sulpa*19 and *sulpa*23 had a large p-value, i.e. close to the borderline of the statistical test.

As with any statistical test, we must also be aware of their weaknesses. For example, the KS test may not give reliable estimates for similar but different cumulative distribution functions since this statistical test is most sensitive near the median of the samples and less sensitive near the tails [146]. As we notice from Fig 5, all our lag time distributions are skewed towards one (very low) extreme of the spectrum. Therefore, we expect that KS test may fail on some of these distributions.

### Three-dimensional reconstructed attractors

We reconstructed three-dimensional attractors based on LFPs from mPFC of ChR2 expressing PV+ interneurons (see Fig 10). We found topologically similar phase space attractors that could be morphed into each other after an appropriate change in delay time (see [102, 103]) for details). The characteristic “8” -shaped attractor (see Fig 10) was also found in the control [102] and cocaine cases [103].

All studies on control data [102], cocaine [103], and this DA receptor antagonists study suggest that the local network could be described by a model with only three variables. Furthermore, all control, cocaine, and DA receptor antagonists trials are classified as similar and placed in the same cluster by the dendrogram method in at least 20% of the cases, regardless of the number of clusters of the dendrogram (see Fig 4c). This overlap suggests that there may be a common, invariant, part of the mathematical model that describes all trials. At the same time, there are some significant difference in the delay times between control and DA receptor antagonists (see Fig 5) that could lead to different phase space dynamics. The differences in the delay times between baseline and DA receptor antagonists, whenever significant, could be interpreted as two different time scales for the two experiments.

We also found that the attractors correctly unfold for a wide range of lag times. Both the attractors unfolded with the autocorrelation-based lag time (see Fig 11a) and with the AMI-based lag time (see Fig 11b) are correctly unfolded. The only difference is that the attractors for unfolded using the very short lag times obtained with the AMI-based method are very narrow along a diagonal.

### Fréchet distance between pairs of attractors

Fechet distance plots shown in Fig 12 provide an intuitive and global understanding of potentially similar time scales between different trials as represented by blue colors. Although the 3D attractors use the lag time to unfold the phase space trajectories (see Fig 10), they lack any temporal dimension. Therefore, Fréchet distance is a measure of only topological similarities between phase space trajectories. In other words, Fréchet distance can tell how close the attractor visits a certain region of the phase space, but not how often or how long the system remains in that region of the phase space. As a result, Fréchet distance offers a complementary view of reconstructed dynamics when compared against lag times, one in which we are interested more in the geometry rather than the dynamics of the phase space. One very good example of the complementarity is *bc*8 attractor that is very similar topologically to both *sulpb*8 and, surprisingly, *sulpa*19 (see Fig 12). From a dynamic point of view, the KS test of lag/correlation times distributions for *bc*8 and *sulpb*8 shows that they were identical (Fig 9b1), but *bc*8 and *sulpa*19 do not have similar lag time distributions. This suggests that different statistical measures capture different aspects of neural activity, some capture the time scale of the dynamics (lag/correlation time) and others capture the topology of the phase space trajectories and the type of accessible states.

### Fourier analysis

The closed phase space trajectories shown in Figs 10 and 11 suggest that spectral (Fourier) analysis may capture the most significant part of the LFP dynamics. We found that Fourier decomposition with more than ten harmonics leaves a white noise residual. Depending on the required accuracy of the model, it is even possible to capture the main trends with less than five Fourier coefficients.

## Conclusions

The activity of the medial prefrontal cortex (mPFC) in 17 mice across six difference combinations of two DA receptor antagonists were investigated. The D1 receptor antagonist SCH 23390 and D2 receptor antagonist sulpiride were used in these experiments either applied **before**, as in *schb*, *sulpb*, and *bothb* trials, or **after**, as in *scha*, *sulpa*, and *botha* trials. The transient phase resetting induced by a light stimulus was removed using the pair correlations between recorded local field potentials (LFPs) [102, 103]. While the improvement in the correlation coefficient is qualitatively apparent from Fig 2, the compact numerical summary in Table 3 shows that the circular shifting improvement exceeds one order of magnitude.

The phase-corrected trials were embedded in a three-dimensional phase space using a delay-embedding method. The main novel results of this study are as follows:

1. We reconstructed the DA receptor antagonist attractor and found that it is three-dimensional.
2. In all trials SCH 23390 after cocaine increases the lag time by about 55% compared to pure cocaine case and when applied before cocaine it decreases the weighted lag time by about 1%. The same trend is preserved when comparing SCH 23390 against baseline, albeit at different percentage changes (see Table 3).
3. Sulpiride after cocaine increases the lag time by about 26% compared to pure cocaine trials, whereas when applied before cocaine it decreases the weighted lag time by about 14%. Sulpiride after cocaine preserves the trend when compared against baseline, although when applied before cocaine produces no change compared to baseline (see Table 3).
4. When both SCH 23390 & sulpiride are applied they seem to reverse the trend observed in the single DA receptor antagonist cases above. For example, when both SCH 23390 & sulpiride were applied after cocaine they seem to decrease the weighted average of lag times by 27%, although the observation is only based on one available mouse. When both SCH 23390 & sulpiride are applied before cocaine its weighted average of lag times increases by 16%. There is no change in the weighted average of the lag times between baseline and both SCH 23390 & sulpiride receptor antagonists case.
5. A spectral model for the LFP recordings with as low as five Fourier coefficients can capture a significant portion of data trends.

To summarize, both SCH 23390 and sulpiride, either applied separately or together, cancel out the effect of cocaine when applied before it (see Table 3). At the same time, both SCH 23390 and sulpiride, either applied separately or together, consistently increase the lag/correlation time of LFP recordings when applied after cocaine.

## Supporting information

S1 Fig Animated three-dimensional reconstructed attractor using autocorrelation-based lag times. The reconstructed attractor for *schb*12 trial #5. The video augments the static picture in Fig 11 and shows that when the attractor is rotated, there are no self-crossings of the phase space trajectories. The autocorrelation-based lag time was 1953 sampling times, i.e. 195.3 ms.
(AVI)

S2 Fig Animated three-dimensional reconstructed attractor using AMI-based lag times. The reconstructed attractor for *schb*12 trial #5. The video augments the static picture in Fig 11 and shows that when the attractor is rotated, there are no self-crossings of the phase space trajectories. The AMI lag time was 205, i.e. 20.5 ms. Although the attractor is correctly unfolded with both correlation- and AMI-based lag times, the very short lag time of AMI method shrinks the attractor along the diagonal and flattens it.
(AVI)

S1 Table Fourier coefficients for signal synthesis. The Fourier amplitudes for *sulpa* mouse #18 were computed for each of the 100m trials with three coefficients (the second and third columns), five coefficients (the fourth and fifth columns), 10 coefficients (the sixth and seventh columns), and 100 coefficients (not shown). The first column represents the integer frequency index *k*. Based on the number of data points and the sampling duration, the actual frequency is *f*_*k*_ = *k*/3 (Hz). Although each trial was synthesized using the largest 3, 5, 10, or 100 Fourier amplitudes, different trials have different combinations of frequencies. For example, when decomposed in three Fourier components, trial #1 requires frequencies *k* = 2, 4, 6 whereas trial # 15 requires frequencies *k* = 2, 4, 8. As a result, for each of the three decompositions in Fourier components, we also show what fraction of the trials required a particular frequency.
(PDF)

## References

[1] Ashby JC, Hitzemann R. Pharmacology of cocaine In: Volkow ND, Swann AC, editors. Cocaine in the Brain. New Brunswick, NJ: Rutgers University Press; 1990 p. 117–134.

[2] Weiss RD, Mirin SM. Subtypes of cocaine abusers. Psychiatric Clinics of North America. 1986;9(3):491–501. 10.1016/S0193-953X(18)30608-7 3774602

[3] Jentsch JD, Olausson P, II RDLG, Taylor JR. Impairments of Reversal Learning and Response Perseveration after Repeated, Intermittent Cocaine Administrations to Monkeys. Neuropsychopharmacologyvolume. 2002;26:183–190. 10.1016/S0893-133X(01)00355-4 11790514

[4] Thompson DM, Moerschbaecher JM. An experimental analysis of the effects of d-amphetamine and cocaine on the acquisition and performance of response chains in monkeys. Journal of the Experimental Analysis of Behavior. 1979;32(3):433–444. 10.1901/jeab.1979.32-433 117072 PMC1332983

[5] Evans E, Wenger G. Effects of drugs of abuse on acquisition of behavioral chains in squirrel monkeys. Psychopharmacology (Berl). 1992;107(1):55–60. 10.1007/BF02244965 1589562

[6] Howell LL, Votaw JR, Goodman MM, Lindsey KP. Cortical activation during cocaine use and extinction in rhesus monkeys. Psychopharmacology. 2009;208(2):191 10.1007/s00213-009-1720-3 19924404 PMC2819208

[7] Fillmore MT, Rush CR, Hays L. Acute effects of oral cocaine on inhibitory control of behavior in humans. Drug and Alcohol Dependence. 2002;67(2):157–167. 10.1016/s0376-8716(02)00062-5 12095665

[8] Garavan H, Kaufman JN, Hester R. Acute effects of cocaine on the neurobiology of cognitive control. Philos Trans R Soc Lond B Biol Sci. 2008;363:3267–3276. 10.1098/rstb.2008.0106 18640911 PMC2607334

[9] Mendelson JH, Mello NK. Management of Cocaine Abuse and Dependence. New England Journal of Medicine. 1996;334(15):965–972. 10.1056/NEJM199604113341507 8596599

[10] Kumar DS, Benedict E, Wu O, Rubin E, Gluck MA, Foltin RW, et al Learning functions in short-term cocaine users. Addictive Behaviors Reports. 2019;9:100169 10.1016/j.abrep.2019.100169 31193767 PMC6542742

[11] Engel AK, Fries P, Kanig P, Brecht M, Singer W. Temporal Binding, Binocular Rivalry, and Consciousness. Consciousness and Cognition. 1999;8(2):128–151. 10.1006/ccog.1999.0389 10447995

[12] Buzsaki G, Draguhn A. Neuronal Oscillations in Cortical Networks. Science. 2004;304(5679):1926–1929. 10.1126/science.1099745 15218136

[13] Jutras MJ, Fries P, Buffalo EA. Gamma-Band Synchronization in the Macaque Hippocampus and Memory Formation. Journal of Neuroscience. 2009;29(40):12521–12531. 10.1523/JNEUROSCI.0640-09.2009 19812327 PMC2785486

[14] Traub R, Jefferys JR, Whittington M. Simulation of Gamma Rhythms in Networks of Interneurons and Pyramidal Cells. Journal of Computational Neuroscience. 1997;4(2):141–150. 10.1023/A:1008839312043 9154520

[15] Feldman ML. Morphology of the neocortical neuron In: Peters A, Jones EG, editors. The Cerebral Cortex. New York: Plenum Press; 1984 p. 123–200.

[16] Bannister AP. Inter- and intra-laminar connections of pyramidal cells in the neocortex. Neuroscience Research. 2005;53(2):95–103. 10.1016/j.neures.2005.06.019 16054257

[17] Sanchez-Vives M, McCormick D. Cellular and network mechanisms of rhythmic recurrent activity in neocortex. Nature Neurosci. 2000;3:1027–1034. 10.1038/79848 11017176

[18] Compte A, Reig R, Descalzo VF, Harvey MA, Puccini GD, Sanchez-Vives MV. Spontaneous High-Frequency (10–80 Hz) Oscillations during Up States in the Cerebral Cortex In Vitro. Journal of Neuroscience. 2008;28(51):13828–13844. 10.1523/JNEUROSCI.2684-08.2008 19091973 PMC6671899

[19] Luczak A, Bartho P, Marguet SL, Buzsaki G, Harris KD. Sequential structure of neocortical spontaneous activity in vivo. Proceedings of the National Academy of Sciences. 2007;104(1):347–352. 10.1073/pnas.0605643104 PMC1765463 17185420

[20] Kana RK, Libero LE, Moore MS. Disrupted cortical connectivity theory as an explanatory model for autism spectrum disorders. Physics of Life Reviews. 2011;8(4):410–437. 10.1016/j.plrev.2011.10.001 22018722

[21] Takahata K, Kato M. Neural mechanism underlying autistic savant and acquired savant syndrome. Brain Nerve. 2008;60(7):861–9. 18646626

[22] Casanova M, Trippe J. Radial cytoarchitecture and patterns of cortical connectivity in autism. Philosophical Transactions of the Royal Society B: Biological Sciences. 2009;364:1433–1436. 10.1098/rstb.2008.0331 PMC2677589 19528027

[23] Galarreta M, Hestrin S. Spike Transmission and Synchrony Detection in Networks of GABAergic Interneurons. Science. 2001;292(5525):2295–2299. 10.1126/science.1061395 11423653

[24] Sultan K, Brown K, Shi SH. Production and organization of neocortical interneurons. Frontiers in Cellular Neuroscience. 2013;7:221 10.3389/fncel.2013.00221 24312011 PMC3836051

[25] Michael A K, Zoe M C. Gamma and beta neural activity evoked during a sensory gating paradigm: Effects of auditory, somatosensory and cross-modal stimulation. Clinical Neuropsychology. 2006;117:2549–2563. 10.1016/j.clinph.2006.08.003 PMC1773003 17008125

[26] Cheng CH, Chan PYS, Niddam DM, Tsai SY, Hsu SC, Liu CY. Sensory gating, inhibition control and gamma oscillations in the human somatosensory cortex. Scientific Reports. 2016;6:20437–47. 10.1038/srep20437 26843358 PMC4740805

[27] Hong L, Summerfelt A, Mitchell BD, McMahon RP, Wonodi I, Buchanan RW, et al Sensory gating endophenotype based on its neural oscillatory pattern and heritability estimate. Archives of General Psychiatry. 2008;65(9):1008–1016. 18762587 10.1001/archpsyc.65.9.1008 PMC2774756

[28] Cardin JA, Carlen M, Meletis K, Knoblich U, Zhang F, Deisseroth K, et al Driving fast-spiking cells induces gamma rhythm and controls sensory responses. Nature. 2009;459:663–7. 10.1038/nature08002 19396156 PMC3655711

[29] Sohal VS, Zhang F, Yizhar O, Deisseroth K. Parvalbumin neurons and gamma rhythms enhance cortical circuit performance. Nature. 2009;459 7247:698–702. 10.1038/nature07991 19396159 PMC3969859

[30] Sohal VS, Zhang F, Yizhar O, Deisseroth K. Insights into Cortical Oscillations Arising from Optogenetic Studies. Biological Psychiatry. 2016;71:1039–1045. 10.1016/j.biopsych.2012.01.024 PMC3361599 22381731

[31] Guidotti A, Auta J, Davis JM, Dong E, Grayson DR, Veldic M, et al GABAergic dysfunction in schizophrenia: new treatment strategies on the horizon. Psychopharmacology. 2005;180(2):191–205. 10.1007/s00213-005-2212-8 15864560

[32] Schmidt M, Mirnics K. Neurodevelopment, GABA System Dysfunction, and Schizophrenia. Neuropsychopharmacology. 2015;40:190–206. 10.1038/npp.2014.95 24759129 PMC4262918

[33] Fuchs EC, Zivkovic AR, Cunningham MO, Middleton S, LeBeau FEN, Bannerman D, et al Recruitment of Parvalbumin-Positive Interneurons Determines Hippocampal Function and Associated Behavior. Neuron. 2007;53(4):591–604. 10.1016/j.neuron.2007.01.031 17296559

[34] Halasy K, Buhl E, Lorinczi Z, Tamas G, Somogyi P. Synaptic target selectivity and input of GABAergic basket and bistratified interneurons in the CA1 area of the rat hippocampus. Hippocampus. 1996;6:306–29. 10.1002/(SICI)1098-1063(1996)6:3<306::AID-HIPO8>3.0.CO;2-K 8841829

[35] Booker SA, Gross A, Althof D, Shigemoto R, Bettler B, Frotscher M, et al Differential GABAB-Receptor-Mediated Effects in Perisomatic- and Dendrite-Targeting Parvalbumin Interneurons. Journal of Neuroscience. 2013;33(18):7961–7974. 10.1523/JNEUROSCI.1186-12.2013 23637187 PMC3814621

[36] Henry TR Markram amd Maria, Yun W, Anirudh G, Gilad S, Caizhi W. Interneurons of the neocortical inhibitory system. Nature Reviews Neuroscience. 2004;5:793–807. 10.1038/nrn1519 15378039

[37] Micheva KD, Wolman D, Mensh BD, Pax E, Buchanan J, Smith SJ, et al A large fraction of neocortical myelin ensheathes axons of local inhibitory neurons. eLife. 2016;5:e15784 10.7554/eLife.15784 27383052 PMC4972537

[38] Melchitzky DS, Lewis DA. Pyramidal Neuron Local Axon Terminals in Monkey Prefrontal Cortex: Differential Targeting of Subclasses of GABA Neurons. Cerebral Cortex. 2003;13(5):452–460. 10.1093/cercor/13.5.452 12679292

[39] Schnitzler A, Gross J. Normal and pathological oscillatory communication in the brain. Nat Rev Neurosci. 2005;6:285–296. 10.1038/nrn1650 15803160

[40] Peter J U, Wolf S. Abnormal neural oscillations and synchrony in schizophrenia. Nature Reviews Neuroscience. 2010;11:100–113. 10.1038/nrn2774 20087360

[41] Levy F. Theories of Autism. Australian & New Zealand Journal of Psychiatry. 2007;41(11):859–868. 10.1080/00048670701634937 17924239

[42] Orekhova EV, Stroganova TA, Nygren G, Tsetlin MM, Posikera IN, Gillberg C, et al Excess of High Frequency Electroencephalogram Oscillations in Boys with Autism. Biological Psychiatry. 2007;62(9):1022–1029. 10.1016/j.biopsych.2006.12.029 17543897

[43] Liddle EB, Price D, Palaniyappan L, Brookes MJ, Robson SE, Hall EL, et al Abnormal salience signaling in schizophrenia: The role of integrative beta oscillations. Human Brain Mapping. 2016;37(4):1361–1374. 10.1002/hbm.23107 26853904 PMC4790909

[44] Lewis DA, Hashimoto T, Volk DW. Cortical inhibitory neurons and schizophrenia. Nat Rev Neurosci. 2005;6(4):312–324. 10.1038/nrn1648 15803162

[45] Lewis DA, Hashimoto T. Deciphering the Disease Process of Schizophrenia: The Contribution of Cortical Gaba Neurons. International Review of Neurobiology. 2007;78:109–131. 10.1016/S0074-7742(06)78004-7 17349859

[46] Ethridge LE, White SP, Mosconi MW, Wang J, Pedapati EV, Erickson CA, et al Neural synchronization deficits linked to cortical hyper-excitability and auditory hypersensitivity in fragile X syndrome. Molecular Autism. 2017;8(1):22 10.1186/s13229-017-0140-1 28596820 PMC5463459

[47] Rotschafer S, Razak K. Auditory Processing in Fragile X Syndrome. Frontiers in Cellular Neuroscience. 2014;8:19 10.3389/fncel.2014.00019 24550778 PMC3912505

[48] Gibson JR, Bartley AF, Hays SA, Huber KM. Imbalance of Neocortical Excitation and Inhibition and Altered UP States Reflect Network Hyperexcitability in the Mouse Model of Fragile X Syndrome. Journal of Neurophysiology. 2008;100(5):2615–2626. 10.1152/jn.90752.2008 18784272 PMC2585391

[49] Contractor A, Klyachko V, Portera-Cailliau C. Altered Neuronal and Circuit Excitability in Fragile X Syndrome. Neuron. 2017;87(4):699–715. 10.1016/j.neuron.2015.06.017 PMC4545495 26291156

[50] Gradinaru V, Thompson KR, Zhang F, Mogri M, Kay K, Schneider MB, et al Targeting and Readout Strategies for Fast Optical Neural Control In Vitro and In Vivo. Journal of Neuroscience. 2007;27(52):14231–14238. 10.1523/JNEUROSCI.3578-07.2007 18160630 PMC6673457

[51] Eleftheriou C, Cesca F, Maragliano L, Benfenati F, Maya-Vetencourt J. Optogenetic Modulation of Intracellular Signalling and Transcription: Focus on Neuronal Plasticity. Journal of Experimental Neuroscience. 2017;11:1179069517703354 10.1177/1179069517703354 28579827 PMC5415353

[52] Kim K, Kim JH, Song YH, Lee SH. Functional dissection of inhibitory microcircuits in the visual cortex. Neuroscience Research. 2017;116:70–76. 10.1016/j.neures.2016.09.003 27633836

[53] Iurilli G, Ghezzi D, Olcese U, Lassi G, Nazzaro C, Tonini R, et al Sound-Driven Synaptic Inhibition in Primary Visual Cortex. Neuron. 2012;73(4):814–828. 10.1016/j.neuron.2011.12.026 22365553 PMC3315003

[54] Wilson NR, Runyan CA, Wang FL, Sur M. Division and subtraction by distinct cortical inhibitory networks in vivo. Nature. 2012;488:343–348. 10.1038/nature11347 22878717 PMC3653570

[55] Liu X, Ramirez S, Pang PT, Puryear CB, Govindarajan A, Deisseroth K, et al Optogenetic stimulation of a hippocampal engram activates fear memory recall. Nature. 2012;484:381–385. 10.1038/nature11028 22441246 PMC3331914

[56] Ramirez S, Tonegawa S, Liu X. Identification and optogenetic manipulation of memory engrams in the hippocampus. Frontiers in Behavioral Neuroscience. 2014;7:226 10.3389/fnbeh.2013.00226 24478647 PMC3894458

[57] Chen Y, Knight ZA. Making sense of the sensory regulation of hunger neurons. BioEssays. 2016;38(4):316–324. 10.1002/bies.201500167 26898524 PMC4899083

[58] Aponte Y, Atasoy D, Sternson SM. AGRP neurons are sufficient to orchestrate feeding behavior rapidly and without training. Nature Neuroscience. 2011;14:351–355. 10.1038/nn.2739 21209617 PMC3049940

[59] Atasoy D, Betley JN, Su HH, Sternson SM. Deconstruction of a neural circuit for hunger. Nature. 2012;488:172–177. 10.1038/nature11270 22801496 PMC3416931

[60] Jennings JH, Rizzi G, Stamatakis AM, Ung RL, Stuber GD. The Inhibitory Circuit Architecture of the Lateral Hypothalamus Orchestrates Feeding. Science. 2013;341(6153):1517–1521. 10.1126/science.1241812 24072922 PMC4131546

[61] Do Monte F, Quirk G, Li B, Penzo M. Retrieving fear memories, as time goes by? Molecular psychiatry. 2016;21:1027–1036. 27217148 10.1038/mp.2016.78 PMC4956525

[62] Haubensak W, Kunwar PS, Cai H, Ciocchi S, Wall NR, Ponnusamy R, et al Genetic dissection of an amygdala microcircuit that gates conditioned fear. Nature. 2010;468:270–276. 10.1038/nature09553 21068836 PMC3597095

[63] Lin D, Boyle MP, Dollar P, Lee H, Lein ES, Perona P, et al Functional identification of an aggression locus in the mouse hypothalamus. Nature. 2011;470(7333):221–226. 10.1038/nature09736 21307935 PMC3075820

[64] Allsop SA, Vander Weele CM, Wichmann R, Tye KM. Optogenetic insights on the relationship between anxiety-related behaviors and social deficits. Frontiers in Behavioral Neuroscience. 2014;8:241 10.3389/fnbeh.2014.00241 25076878 PMC4099964

[65] Tye KM, Prakash R, Kim SY, Fenno LE, Grosenick L, Zarabi H, et al Amygdala circuitry mediating reversible and bidirectional control of anxiety. Nature. 2011;471(7338):358–362. 10.1038/nature09820 21389985 PMC3154022

[66] Tye KM, Mirzabekov JJ, Warden MR, Ferenczi EA, Tsai HC, Finkelstein J, et al Dopamine neurons modulate neural encoding and expression of depression-related behaviour. Nature. 2013;493(7433):537–541. 10.1038/nature11740 23235822 PMC4160519

[67] Gradinaru V, Mogri M, Thompson KR, Henderson JM, Deisseroth K. Optical Deconstruction of Parkinsonian Neural Circuitry. Science. 2009;324(5925):354–359. 10.1126/science.1167093 19299587 PMC6744370

[68] Kravitz AV, Freeze BS, Parker PRL, Kay K, Thwin MT, Deisseroth K, et al Regulation of parkinsonian motor behaviours by optogenetic control of basal ganglia circuitry. Nature. 2010;466:622–626. 10.1038/nature09159 20613723 PMC3552484

[69] Wykes RC, Kullmann DM, Pavlov I, Magloire V. Optogenetic approaches to treat epilepsy. Journal of Neuroscience Methods. 2016;260:215–220. 10.1016/j.jneumeth.2015.06.004 26072246

[70] Peng Z, Zhang N, Wei W, Huang CS, Cetina Y, Otis TS, et al A Reorganized GABAergic Circuit in a Model of Epilepsy: Evidence from Optogenetic Labeling and Stimulation of Somatostatin Interneurons. Journal of Neuroscience. 2013;33(36):14392–14405. 10.1523/JNEUROSCI.2045-13.2013 24005292 PMC3761049

[71] Kokaia M, Andersson M, Ledri M. An optogenetic approach in epilepsy. Neuropharmacology. 2013;69:89–95. 10.1016/j.neuropharm.2012.05.049 22698957

[72] Paz JT, Davidson TJ, Frechette ES, Delord B, Parada I, Peng K, et al Closed-loop optogenetic control of thalamus as a tool for interrupting seizures after cortical injury. Nat Neurosci. 2013;16(1):64–70. 10.1038/nn.3269 23143518 PMC3700812

[73] Gelder RNV. Photochemical approaches to vision restoration. Vision Research. 2015;111:134–141. 10.1016/j.visres.2015.02.001 25680758 PMC4444397

[74] Busskamp V, Duebel J, Balya D, Fradot M, Viney TJ, Siegert S, et al Genetic Reactivation of Cone Photoreceptors Restores Visual Responses in Retinitis Pigmentosa. Science. 2010;329(5990):413–417. 10.1126/science.1190897 20576849

[75] Lagali PS, Balya D, Awatramani GB, Munch TA, Kim DS, Busskamp V, et al Light-activated channels targeted to ON bipolar cells restore visual function in retinal degeneration. Nat Neurosci. 2008;11:667–675. 10.1038/nn.2117 18432197

[76] Rivnay J, Wang H, Fenno L, Deisseroth K, Malliaras G. Next-generation probes, particles, and proteins for neural interfacing. Science Advances. 2017;3:e1601649 10.1126/sciadv.1601649 28630894 PMC5466371

[77] Ritz M, Lamb R, Goldberg S, Kuhar M. Cocaine receptors on dopamine transporters are related to self-administration of cocaine. Science. 1987;237(4819):1219–1223. 2820058 10.1126/science.2820058

[78] Weed MR, Woolverton WL. The reinforcing effects of dopamine D1 receptor agonists in rhesus monkeys. Journal of Pharmacology and Experimental Therapeutics. 1995;275(3):1367–1374. 8531104

[79] Iravani MM, Asari D, Patel JC, Wieczorek WJ, Kruk ZL. Direct effects of 3,4-methylenedioxymethamphetamine (MDMA) on serotonin or dopamine release and uptake in the caudate putamen, nucleus accumbens, substantia nigra pars reticulata, and the dorsal raphé nucleus slices. Synapse. 2000;364:275–85. 10.1002/(SICI)1098-2396(20000615)36:4<275::AID-SYN4>3.0.CO;2-# 10819905

[80] Dunlap LE, Andrews AM, Olson DE. Dark Classics in Chemical Neuroscience: 3,4-Methylenedioxymethamphetamine. ACS chemical neuroscience. 2018;9:2408–2427. 10.1021/acschemneuro.8b00155 30001118 PMC6197894

[81] Koob GF. Drugs of abuse: anatomy, pharmacology and function of reward pathways. Trends in Pharmacological Sciences. 1992;13:177–184. 10.1016/0165-6147(92)90060-j 1604710

[82] Chiara GD. Drug addiction as dopamine-dependent associative learning disorder. European Journal of Pharmacology. 1999;375(1):13–30. 10.1016/s0014-2999(99)00372-6 10443561

[83] Bergman J, Kamien JB, Spealman RD. Antagonism of cocaine self-administration by selective dopamine D1 and D2 antagonists. Behavioural pharmacology. 1990;1:355–363. 10.1097/00008877-199000140-00009 11175420

[84] Watkins SS, Epping-Jordan MP, Koob GF, Markou A. Blockade of Nicotine Self-Administration with Nicotinic Antagonists in Rats. Pharmacology Biochemistry and Behavior. 1999;62(4):743–751. 10.1016/S0091-3057(98)00226-3 10208381

[85] Daniela E, Brennan K, Gittings D, Hely L, Schenk S. Effect of SCH 23390 on (±)-3,4-methylenedioxymethamphetamine hyperactivity and self-administration in rats. Pharmacology Biochemistry and Behavior. 2004;77(4):745–750. 10.1016/j.pbb.2004.01.008 15099919

[86] Wise R, Murray A, Bozarth M. Bromocriptine self-administration and bromocriptine-reinstatement of cocaine-trained and heroin-trained lever pressing in rats. Psychopharmacology (Berl). 1990;100(3):355–360. 10.1007/BF02244606 2315433

[87] Spealman RD, Barrett-Larimore RL, Rowlett JK, Platt DM, Khroyan TV. Pharmacological and Environmental Determinants of Relapse to Cocaine-Seeking Behavior. Pharmacology Biochemistry and Behavior. 1999;64(2):327–336. 10.1016/S0091-3057(99)00049-0 10515309

[88] Reiner DJ, Fredriksson I, Lofaro OM, Bossert JM, Shaham Y. Relapse to opioid seeking in rat models: behavior, pharmacology and circuits. Neuropsychopharmacology. 2019;44(3):465–477. 10.1038/s41386-018-0234-2 30293087 PMC6333846

[89] Farrell MR, Schoch H, Mahler SV. Modeling cocaine relapse in rodents: Behavioral considerations and circuit mechanisms. Progress in Neuro-Psychopharmacology and Biological Psychiatry. 2018;87:33–47. 10.1016/j.pnpbp.2018.01.002 29305936 PMC6034989

[90] Self DW, Barnhart WJ, Lehman DA, Nestler EJ. Opposite Modulation of Cocaine-Seeking Behavior by D1- and D2-Like Dopamine Receptor Agonists. Science. 1996;271(5255):1586–1589. 10.1126/science.271.5255.1586 8599115

[91] Mello N, Negus S. Preclinical Evaluation of Pharmacotherapies for Treatment of Cocaine and Opioid Abuse Using Drug Self-Administration Procedures. Neuropsychopharmacology. 1996;14:375–424. 10.1016/0893-133X(95)00274-H 8726752

[92] Haney M, Ward AS, Foltin RW, Fischman MW. Effects of ecopipam, a selective dopamine D1 antagonist, on smoked cocaine self-administration by humans. Psychopharmacology. 2001;155(4):330–337. 10.1007/s002130100725 11441422

[93] Romach MK, Glue P, Kampman K, Kaplan HL, Somer GR, Poole S, et al Attenuation of the Euphoric Effects of Cocaine by the Dopamine D1/D5 Antagonist Ecopipam (SCH 39166). Archives of General Psychiatry. 1999;56(12):1101–1106. 10591286 10.1001/archpsyc.56.12.1101

[94] Jackson DM, Westlind-Danielsson A. Dopamine receptors: Molecular biology, biochemistry and behavioural aspects. Pharmacology & Therapeutics. 1994;64(2):291–370. 10.1016/0163-7258(94)90041-8 7878079

[95] Rangel-Barajas C, Coronel I, Floran B. Dopamine Receptors and Neurodegeneration. Aging and disease. 2015;6(5):349–368. 10.14336/AD.2015.0330 26425390 PMC4567218

[96] Kebabian JW, Calne DB. Multiple receptors for dopamine. Naure. 1979;277:93–96. 10.1038/277093a0 215920

[97] Mishra A, Singh S, Shukla S. Physiological and Functional Basis of Dopamine Receptors and Their Role in Neurogenesis: Possible Implication for Parkinson’s disease. Journal of experimental neuroscience. 2018;12. 10.1177/1179069518779829 PMC5985548 29899667

[98] Self DW, Stein L. The D1 agonists SKF 82958 and SKF 77434 are self-administered by rats. Brain Research. 1992;582(2):349–352. 10.1016/0006-8993(92)90155-3 1356585

[99] Caine SB, Koob GF. Effects of dopamine D-1 and D-2 antagonists on cocaine self-administration under different schedules of reinforcement in the rat. Journal of Pharmacology and Experimental Therapeutics. 1994;270(1):209–218. 8035317

[100] Khroyan TV, Barrett-Larimore RL, Rowlett JK, Spealman RD. Dopamine D1- and D2-Like Receptor Mechanisms in Relapse to Cocaine-Seeking Behavior: Effects of Selective Antagonists and Agonists. Journal of Pharmacology and Experimental Therapeutics. 2000;294(2):680–687. 10900248

[101] Caine SB, Negus SS, Mello NK. Effects of dopamine D1-like and D2-like agonists on cocaine self-administration in rhesus monkeys: rapid assessment of cocaine dose-effect functions. Psychopharmacology. 2000;148(1):41–51. 10.1007/s002130050023 10663416

[102] Oprisan SA, Lynn PE, Tompa T, Lavin A. Low-dimensional attractor for neural activity from local field potentials in optogenetic mice. Frontiers in Computational Neuroscience. 2015;9:125 10.3389/fncom.2015.00125 26483665 PMC4591433

[103] Oprisan SA, Imperatore J, Helms J, Tompa T, Lavin A. Cocaine-Induced Changes in Low-Dimensional Attractors of Local Field Potentials in Optogenetic Mice. Frontiers in Computational Neuroscience. 2018;12:2 10.3389/fncom.2018.00002 29445337 PMC5797774

[104] Oprisan SA. All Phase Resetting Curves Are Bimodal, but Some Are More Bimodal Than Others. ISRN Computational Biology. 2013;2013(Article ID 230571):1–11. 10.1155/2013/230571

[105] Oprisan SA. A Consistent Definition of Phase Resetting Using Hilbert Transform. International Scholarly Research Notices Computational Biology. 2017;2017(Article ID 5865101):10. 10.1155/2017/5865101 PMC5434474 28553658

[106] Oprisan SA, Canavier CC. The influence of limit cycle topology on the phase resetting curve. Neural Computation. 2002;14:1027–2002. 10.1162/089976602753633376 11972906

[107] Oprisan SA, Thirumalai V, Canavier CC. Dynamics from a time series: Can we extract the phase resetting curve from a time series? Biophysical Journal. 2003;84:2919–2928. 10.1016/S0006-3495(03)70019-8 12719224 PMC1302855

[108] Dilgen J, Tompa T, Saggu S, Naselaris T, Lavin A. Optogenetically evoked gamma oscillations are disturbed by cocaine administration. Frontiers in Cellular Neuroscience. 2013;7:213 10.3389/fncel.2013.00213 24376397 PMC3841795

[109] Saraçli S, Doğan N, Doğan İ. Comparison of hierarchical cluster analysis methods by cophenetic correlation. Journal of Inequalities and Applications. 2013;2013(1):203 10.1186/1029-242X-2013-203

[110] Hill T, Lewicki P, editors. Statistics: Methods and Applications. Tulksa, OK: StatSoft, Inc; 2005.

[111] Xu P. Differential phase space reconstructed for chaotic time series. Applied Mathematical Modelling. 2009;33(2):999–1013. 10.1016/j.apm.2007.12.021

[112] Takens F. Detecting strange attractors in turbulence In: Rand D, Young LS, editors. Dynamical Systems and Turbulence, Warwick 1980. vol. 898 of Lecture Notes in Mathematics. Springer Berlin Heidelberg; 1981 p. 366–381.

[113] Diks C, van Houwelingen JC, Takens F, DeGoede J. Reversibility as a criterion for discriminating time series. Phys Lett A. 1995;201:221–228. 10.1016/0375-9601(95)00239-Y

[114] Packard NH, Crutchfield JP, Farmer JD, Shaw RS. Geometry from a Time Series. Phys Rev Lett. 1980;45:712–716. 10.1103/PhysRevLett.45.712

[115] Whitney H. Differentiable Manifolds. Annals of Mathematics. 1936;37(3):645–680. 10.2307/1968482

[116] Mañé R. On the dimension of the compact invariant sets of certain non-linear maps In: Rand D, Young LS, editors. Dynamical Systems and Turbulence, Warwick 1980. Berlin, Heidelberg: Springer Berlin Heidelberg; 1981 p. 230–242.

[117] Casdagli M, Eubank S, Farmer JD, Gibson J. State Space Reconstruction in the Presence of Noise. Phys D. 1991;51(1-3):52–98. 10.1016/0167-2789(91)90222-U

[118] Zeng X, Eykholt R, Pielke RA. Estimating the Lyapunov-exponent spectrum from short time series of low precision. Phys Rev Lett. 1991;66:3229–3232. 10.1103/PhysRevLett.66.3229 10043734

[119] Schiff SJ, Chang T. Differentiation of linearly correlated noise from chaos in a biologic system using surrogate data. Biological Cybernetics. 1992;67(5):387–393. 10.1007/bf00200982 1391112

[120] Schuster HG, Just W, editors. Deterministic Chaos: An Introduction, 4th, Revised and Enlarged Edition Weinheim: WILEY-VCH Verlag GmbH and Co. KGaA; 2005.

[121] King GP, Jones R, Broomhead DS. Phase portraits from a time series: A singular system approach. Nuclear Physics B—Proceedings Supplements. 1987;2:379–390. 10.1016/0920-5632(87)90029-6

[122] Holzfuss J, Mayer-Kress G. An Approach to Error-Estimation in the Application of Dimension Algorithms. In: Mayer-Kress G, editor. Dimensions and Entropies in Chaotic Systems. vol. 32 of Springer Series in Synergetics; 1986 p. 114–122. 10.1007/978-3-642-71001-8_15

[123] Fraser AM, Swinney HL. Independent coordinates for strange attractors from mutual information. Phys Rev A. 1986;33:1134–1140. 10.1103/PhysRevA.33.1134 9896728

[124] Hegger R, Kantz H, Schreiber T. Practical implementation of nonlinear time series methods: The TISEAN package. Chaos. 1999;9:413–435. 10.1063/1.166424 12779839

[125] Kantz H, Schreiber T, editors. Non-linear Time Series Analysis. Cambridge: Cambridge University Press; 1997.

[126] Abarbanel HDI, editor. Analysis of Observed Chaotic Data. New York: Springer; 1996.

[127] Kennel MB, Brown R, Abarbanel HDI. Determining embedding dimension for phase-space reconstruction using a geometrical construction. Phys Rev A. 1992;45:3403–3411. 10.1103/physreva.45.3403 9907388

[128] Sen AK, Litak G, Syta A. Cutting process dynamics by nonlinear time series and wavelet analysis. Chaos: An Interdisciplinary Journal of Nonlinear Science. 2007;17(2). 10.1063/1.2749329 17614687

[129] Kugiumtzis D. Surrogate Data Test on Time Series In: Soofi A, Cao L, editors. Modelling and Forecasting Financial Data. vol. 2 of Studies in Computational Finance. Springer US; 2002 p. 267–282.

[130] Grassberger P. Evidence for climatic attractors. Nature. 1987;362:524 10.1038/326524a0

[131] Theiler J. Estimating fractal dimension. J Opt Soc Am A. 1990;7(6):1055–1073. 10.1364/JOSAA.7.001055

[132] Theiler J, Eubank S, Longtin A, Galdrikian B, Farmer JD. Testing for nonlinearity in time series: the method of surrogate data. Physica D. 1992;58(58):77–94. 10.1016/0167-2789(92)90102-S

[133] Frechet M. Sur quelques points du calcul fonctionnel. Rendiconti del Circolo Mathematico di Palermo. 1906;22:1–74. 10.1007/BF03018603

[134] Eiter T, Mannila H. Computing discrete Frechet distance. Technical University of Vienna and University of Helsinki; 1994.

[135] Alt H, Godau M. Computiong the Frechet distance between towo polgonal curves. International Journal of Computational Geometry & Applications. 1995;05(01n02):75–91. 10.1142/S0218195995000064

[136] Danziger A. Discrete Frechet Distance; 2013. https://www.mathworks.com/matlabcentral/fileexchange/31922-discrete-frechet-distance?focused=3785717&tab=function&requestedDomain=www.mathworks.com.

[137] Howell KB. Principles of Fourier Analysis Textbooks in Mathematics. CRC Press; 2001 Available from: https://books.google.com/books?id=Q5HMBQAAQBAJ.

[138] Stein EM, Shakarchi R. Fourier Analysis: An Introduction Princeton Lectures in Analysis Series. Princeton University Press; 2003.

[139] Osorio I, Frei MG. Seizure abatement with single DC pulses: ias phase resetting at play? International Journal of Neural Systems. 2009;19(03):149–156. 19575505 10.1142/S0129065709001926

[140] Parastarfeizabadi M, Kouzani AZ. Advances in closed-loop deep brain stimulation devices. Journal of NeuroEngineering and Rehabilitation. 2017;14(1):79 10.1186/s12984-017-0295-1 28800738 PMC5553781

[141] Tass PA. Stochastic Phase Resetting: A Theory for Deep Brain Stimulation. Progress of Theoretical Physics Supplement. 2000;139:301–313. 10.1143/PTPS.139.301

[142] Tass PA. A model of desynchronizing deep brain stimulation with a demand-controlled coordinated reset of neural subpopulations. Biological Cybernetics. 2003;89(2):81–88. 10.1007/s00422-003-0425-7 12905037

[143] Oprisan SA, Prinz A, Canavier CC. Phase resetting and phase locking in hybrid circuits of one model and one biological neuron. Biophysical Journal. 2004;87:2283–2298. 10.1529/biophysj.104.046193 15454430 PMC1304653

[144] Oprisan SA, Austin DI. A Generalized Phase Resetting Method for Phase-Locked Modes Prediction. PLoS ONE. 2017;12(3):e0174304 10.1371/journal.pone.0174304 28323894 PMC5360347

[145] Chakravarti IM, Laha RG, Roy J. Handbook of methods of applied statistics No. v. 1 in Wiley series in probability and mathematical statistics. Wiley; 1967.

[146] Steinskog DJ, Tjostheim DB, Kvamsto NG. A Cautionary Note on the Use of the Kolmogorov—Smirnov Test for Normality. Monthly Weather Review. 2007;135(3):1151–1157. 10.1175/MWR3326.1

