# Supplementary material for: Dopamine receptor antagonists effects on low-dimensional attractors of local field potentials in optogenetic mice
Source: PLoS One. 2019 Oct 16;14(10):e0223469. doi: 10.1371/journal.pone.0223469 (PMC6795423; doi:10.1371/journal.pone.0223469)
Supplement: S1 Table — The Fourier amplitudes for sulpa mouse #18 were computed for each of the 100m trials with three coefficients (the second and third columns), five coefficients (the fourth and fifth columns), 10 coefficients (the sixth and seventh columns), and 100 coefficients (not shown). The first column represents the integer frequency index k. Based on the number of data points and the sampling duration, the actual frequency is fk = k/3 (Hz). Although each trial was synthesized using the largest 3, 5, 10, or 100 Fourier amplitudes, different trials have different combinations of frequencies. For example, when decomposed in three Fourier components, trial #1 requires frequencies k = 2, 4, 6 whereas trial # 15 requires frequencies k = 2, 4, 8. As a result, for each of the three decompositions in Fourier components, we also show what fraction of the trials required a particular frequency. (PDF) [file pone.0223469.s003.pdf]

Table 1. Fourier coefficients for signal synthesis. The Fourier amplitudes for *sulpa* mouse #18 were computed for each of the 100m trials with three coefficients (the second and third columns), five coefficients (the fourth and fifth columns), 10 coefficients (the sixth and seventh columns), and 100 coefficients (not shown). The first column represents the integer frequency index k. Based on the number of data points and the sampling duration, the actual frequency is $f_k = k/3$ (Hz). Although each trial was synthesized using the largest 3, 5, 10, or 100 Fourier amplitudes, different trials have different combinations of frequencies. For example, when decomposed in three Fourier components, trial #1 requires frequencies $k = 2, 4, 6$ whereas trial # 15 requires frequencies $k = 2, 4, 8$. As a result, for each of the three decompositions in Fourier components, we also show what fraction of the trials required a particular frequency.

k	% trials (3 coeff.)	A_k (3 coeff.)	% trials (5 coeff.)	A_k (5 coeff.)	% trials (10 coeff.)	A_k (10 coeff.)
4	31	0.059 ± 0.038	23	0.059 ± 0.041	12.9	0.059 ± 0.037
6	20	0.091 ± 0.068	16	0.083 ± 0.069	9.1	0.083 ± 0.067
8	15	0.125 ± 0.090	11	0.125 ± 0.097	6.1	0.125 ± 0.090
10	15	0.125 ± 0.092	11	0.125 ± 0.10	6.1	0.125 ± 0.10
12	7	0.25 ± 0.13	7	0.20 ± 0.17	3.8	0.20 ± 0.19
14	5	0.33 ± 0.23	7	0.20 ± 0.20	3.8	0.20 ± 0.22
16	7	0.25 ± 0.17	7	0.20 ± 0.15	4.5	0.17 ± 0.15
18			4	0.33 ± 0.19	3.0	0.25 ± 0.17
20			3	0.50 ± 0.24	2.2	0.33 ± 0.25
22			4	0.33 ± 0.25	2.2	0.33 ± 0.27
24			3	0.50 ± 0	2.2	0.33 ± 0.22
26			1	1	3.0	0.25 ± 0.25
28			1	1	1.5	0.50 ± 0.031
30			3	0.5	2.2	0.33 ± 0.26
32					2.2	0.33 ± 0.35
34					1.5	0.50 ± 0.24
36					1.5	0.50 ± 0.079
38					1.5	0.50 ± 0.10
40					1.5	0.50 ± 0.064
42					0.8	1
44					1.5	0.5
46					1.5	0.50 ± 0.24
48					1.5	0.50 ± 0.24
50					0.8	1
52					1.5	0.5
54					1.5	0.50 ± 0.35
56					1.5	0.5
58					1.5	0.50 ± 0.24
60					1.5	0.5
62					1.5	0.5
64					1.5	0.5
68					0.8	1
70					0.8	1
74					1.5	0.50 ± 0.35
86					0.8	1
88					0.8	1
92					0.8	1
94					0.8	1
108					0.8	1
118					0.8	1
122					0.8	1
132					0.8	1
136					0.8	1
148					0.8	1
